# Predictive Value of Ov16 Antibody Prevalence in Different Subpopulations for Elimination of African Onchocerciasis

**DOI:** 10.1093/aje/kwz109

**Published:** 2019-05-07

**Authors:** Luc E Coffeng, Wilma A Stolk, Allison Golden, Tala de los Santos, Gonzalo J Domingo, Sake J de Vlas

**Affiliations:** 1 Department of Public Health, Erasmus MC, University Medical Center Rotterdam, Rotterdam, the Netherlands; 2 PATH, Seattle, Washington

**Keywords:** agent-based modeling, antibodies, disease elimination, infectious disease transmission, mass drug administration, onchocerciasis, predictive value of tests

## Abstract

The World Health Organization currently recommends assessing elimination of onchocerciasis by testing whether Ov16 antibody prevalence in children aged 0–9 years is below 0.1%. However, the certainty of evidence for this recommendation is considered to be low. We used the established ONCHOSIM model to investigate the predictive value of different Ov16-antibody prevalence thresholds in various age groups for elimination of onchocerciasis in a variety of endemic settings and for various mass drug administration scenarios. According to our simulations, the predictive value of Ov16 antibody prevalence for elimination depends highly on the precontrol epidemiologic situation, history of mass drug administration, the age group that is sampled, and the chosen Ov16-antibody prevalence threshold. The Ov16 antibody prevalence in children aged 5–14 years performs best in predicting elimination. Appropriate threshold values for this age group start at 2.0% for very highly endemic areas; for lower-endemic areas, even higher threshold values are safe to use. Guidelines can be improved by sampling school-aged children, which also is operationally more feasible than targeting children under age 10 years. The use of higher threshold values allows sampling of substantially fewer children. Further improvement can be achieved by taking a differentiated sampling approach based on precontrol endemicity.

Onchocerciasis, a parasitic worm infection also known as river blindness, is targeted for elimination in Africa by 2025 through annual or semiannual mass drug administration (MDA) with ivermectin (1, 2). This goal requires careful monitoring and evaluation of ongoing transmission and recrudescence of infection. The traditional parasitological method of counting microfilariae (mf) in skin snips (superficial skin biopsies) is not sensitive enough when prevalence and intensity of infection become very low after prolonged control (3). Therefore, the World Health Organization recommends basing the decision to stop MDA on 2 more-sensitive techniques to detect ongoing or returning transmission: pool screening of the vector blackflies for presence of parasite DNA, and serological surveys among children under the age of 10 years for presence of Ov16 antibodies (4). The current recommendation is that the prevalence of Ov16 antibodies should be under 0.1% before considering stopping MDA, which requires sampling several thousand children. The certainty of evidence for this recommendation is considered low (4).

An enzyme-linked immunosorbent assay for anti-Ov16 immunoglobulin G4 (IgG4) has been used for monitoring and evaluation of onchocerciasis control in Latin America (5–12) and Africa (13–19), and this is the technique currently recommended by the World Health Organization to evaluate anti-Ov16 IgG4 (4). A standardized point-of-contact test has been also developed, using lateral flow strips for detection of anti-Ov16 IgG4, optionally combined within a biplex for simultaneous detection of Wb123 antibodies against *Wuchereria bancrofti* (20, 21). Further validation of this point-of-contact test is still required for it to possibly replace the enzyme-linked immunosorbent assay as the technique recommended by the World Health Organization (4). Recently, we predicted how serological tests in general would perform in African settings, and we concluded that test results—regardless of technique—strongly depend on the precontrol endemicity, meaning that a one-size-fits-all protocol might lead to stopping MDA too soon in high-endemic settings and later than necessary in low-endemic ones (22).

We investigated the predictive value of Ov16 antibody prevalence for elimination of onchocerciasis under different diagnostic criteria and sampling strategies. We used the established ONCHOSIM model to simulate a variety of endemic settings and MDA scenarios and to calculate the probability of elimination for a range of threshold values of the Ov16 antibody prevalence in various age groups. Based on this, we provide more tailored guidelines for the use of Ov16 antibody prevalence as an indicator for elimination of African onchocerciasis.

## METHODS

To evaluate the predictive value of Ov16 antibody prevalence in assessing elimination of African onchocerciasis, we used ONCHOSIM (23), an individual-based model for transmission and control of onchocerciasis that has been extensively used to support decision making in onchocerciasis-control programs in Africa (24–34). In Web Appendix 1 (available at https://academic.oup.com/aje), we describe how transmission and control are modeled with ONCHOSIM.

For this study, we assumed that an individual’s Ov16 serostatus is a binary variable, similar to the IgG4-based Ov16 antibody rapid diagnostic test: Individuals are either seropositive or seronegative; degrees of antibody levels are not considered. Because it is not exactly known how seroconversion is triggered and how long it takes after the trigger for an individual to become seropositive, we previously considered 3 alternative hypotheses (22). Given that some studies suggest that antibodies can be detectable before skin mf (35, 36) (although this concerns total IgG antibodies and not the more specific IgG4 used for Ov16 testing), in the present study we assumed that Ov16 seroconversion occurs when the first male or female worm in the human body has matured. Seroconversion can thus precede the occurrence of mf in the skin if a single worm or single-sex infection triggers seroconversion, but it can also coincide with the appearance of mf when a male-female worm pair is present. Antibody positivity in combination with mf negativity might also occur due to false-negative mf tests at low mf densities or after clearance of infection. We further assumed 2 extremes concerning seroreversion: seropositivity is either lifelong (i.e., no seroreversion) or lasts until the last adult female worm in an individual has died (i.e., instant seroreversion). Some degree of seropositivity loss was recently demonstrated in a study in Togo, but the study did not allow for careful quantification of this process (37). Last, because the risk that misclassification of an individual’s serostatus might vary between different types of Ov16 antibody tests, we performed a reference analysis assuming that we knew the exact serostatus of each individual. In Web Appendix 2, we explain how the reader can interpret our results accounting for misclassification of serostatus.

**Figure 1.**
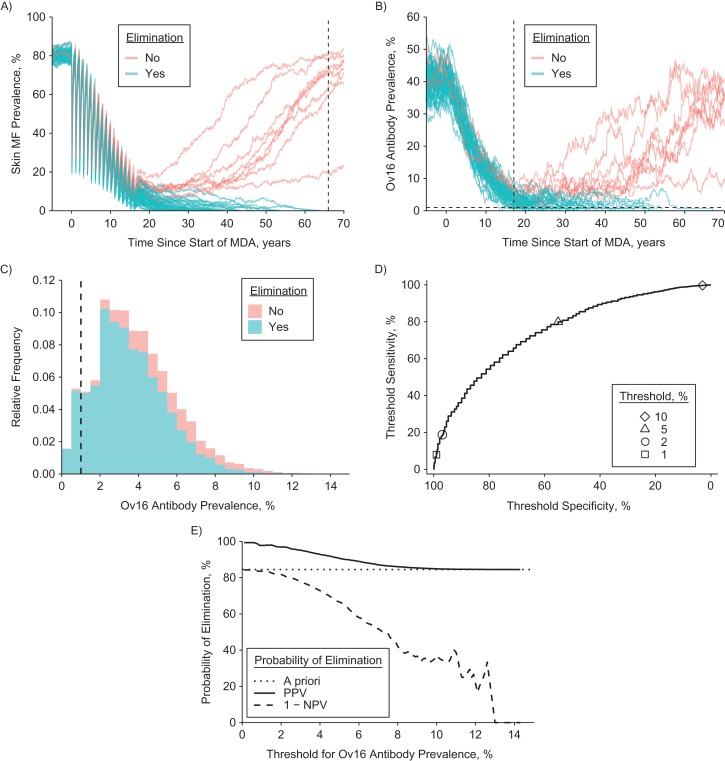
Overview of analytical steps to evaluate the predictive value of Ov16 antibody prevalence. The figure considers the procedure for a single scenario, which for illustrative purposes we take to represent a setting with precontrol community microfilarial load of 55 microfilariae (mf) per skin snip (equivalent to skin mf prevalence of ~80%) and 6 years of annual mass drug administration (MDA). In this example, Ov16 positivity is assumed to be lifelong; patterns are qualitatively similar when based on the assumption of instant seroreversion. Note that the jagged appearance of the curves is due to the stochastic nature of the simulations. A) Using ONCHOSIM, we first predict trends in the prevalence of skin mf up to 50 years after the last MDA round and classify repeated simulations (graph lines) as resulting in either elimination or ongoing transmission. Here, we only show 50 of the 10,000 repeated runs performed, of which 8 did not result in elimination. B) From exactly the same repeated simulation runs, we extracted the predicted prevalence of Ov16 antibodies in selected age groups (example here: children of aged <10 years). C) We then extracted the frequency distribution of Ov16 antibody prevalence across repeated simulations, 1 year after the last MDA round, and stratified it by simulation outcome in terms of elimination (colored areas) and a chosen threshold for Ov16 antibody prevalence (vertical dashed line, here set at 1%). See Table 1 for the resulting classification of simulations and the calculation of sensitivity, specificity, and positive and negative predictive value of Ov16 antibody prevalence. D) We subsequently plotted threshold sensitivity versus threshold specificity for the range of threshold values in a receiver operator characteristic curve. E) Finally, we plotted positive predictive value (PPV, probability of elimination when Ov16 prevalence is under the threshold) and 1 minus negative predictive value (NPV, probability of elimination when Ov16 antibody prevalence is above the threshold) against the range of threshold values (horizontal axis). Note that when interpreting this figure, the threshold value (*x*-axis) is decided on before observing the Ov16 antibody prevalence (i.e., the *x*-axis represents the predefined threshold and not the observed Ov16 prevalence).

Using ONCHOSIM, we simulated trends in prevalence of infection and Ov16 antibody prevalence for 750 scenarios related to precontrol endemicity (5 levels; details provided in Web Appendix 3) and MDA frequency (annual vs. semiannual), coverage (60%, 70%, or 80%), and duration (1–25 years). For each scenario, we evaluated the Ov16 prevalences in various age groups 1 year after the last MDA round and compared these with the simulation outcome in terms of ongoing transmission versus elimination (nonzero vs. zero mf prevalence 50 years after the last MDA round). Because ONCHOSIM is a stochastic simulation model (i.e., predictions vary because events within the simulation occur in a random fashion), each scenario was simulated 10,000 times, yielding a sample of 10,000 draws from the joint distribution of Ov16 prevalence and the simulation outcome in terms of ongoing transmission versus elimination. See Figure 1A–C for an illustration. For each of the 750 scenarios and a range of Ov16 antibody prevalence thresholds (0%–100% in 0.1% steps), we categorized the 10,000 repeated Ov16 prevalence outcomes (Table 1) as true positive (Ov16 antibody prevalence under the threshold value and simulation resulting in elimination), false negative (above the threshold value but still resulting in elimination), false positive (under the threshold value but resulting in ongoing transmission), or true negative (above the threshold and resulting in ongoing transmission). We used receiver operating characteristic curves to visualize the sensitivity and specificity of Ov16 antibody prevalence thresholds for the prediction of elimination under the range of potential threshold values (Figure 1D), which we will from here on refer to as threshold sensitivity and specificity (as opposed to test sensitivity and specificity for detection of antibodies). This whole process was repeated for Ov16 antibody prevalence in several age groups within the simulated population, in years: 0–4, 5–9, 10–14, and 15–19, as well as the broader categories of age 0–9 (current target age group) and 5–14 (school-age children) years. Receiver operating characteristic curves for different age groups were compared to identify which age groups provided most information about prospects of elimination. We further estimated the probability of elimination if the Ov16 antibody prevalence is under the threshold (positive predictive value or PPV) or above (1 minus the negative predictive value). See Figure 1E for an illustration. PPV curves were used to assess the predictive value of threshold values across different endemic settings and MDA histories.

**Table 1.** Example Classification of 10,000 Repeated Simulations for Calculation of the Predictive Value of Ov16 Antibody Prevalence for Elimination of Onchocerciasis

Simulation Outcome 50 Years After Last Round of MDA^a^	Ov16 Antibody Prevalence 1 Year After Last Round of MDA^b^		Total
	<1%	≥1%	
Ongoing transmission	15 (FP)	1,534 (TN)	1,549
Elimination^c^	670 (TP)	7,781 (FN)	8,451
Total	685	9,315	10,000

Abbreviations: FN, false negative; FP, false positive; MDA, mass drug administration; mf, microfilaria(e); TN, true negative; TP, true positive.

^a^ Simulation scenario shown in Figure 1.

^b^ Example calculation of sensitivity, specificity, and positive and negative predictive value of a 1% threshold for Ov16 seroprevalence for prediction of elimination: sensitivity = TP/(TP + FN) = 7.9%; specificity = TN/(TN + FP) = 99.0%; positive predictive value = TP/(TP + FP) = 97.8%; negative predictive value = TN/(TN + FN) = 16.5%.

^c^ Zero mf prevalence 50 years after the last MDA round, which occurred in (TP + FN)/(TP + FN + TN + FN) = 84.5% of the 10,000 repeated simulations.

## RESULTS

**Figure 2.**
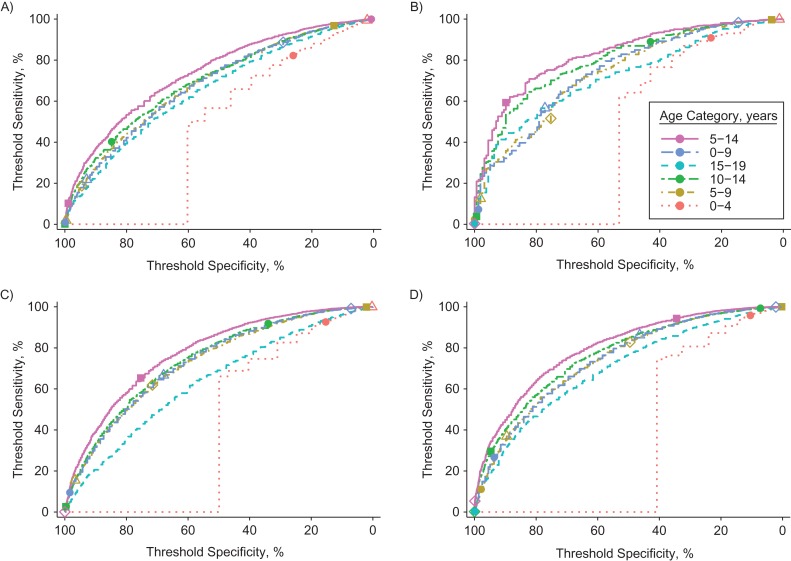
Receiver operating characteristic curve for Ov16 antibody prevalence as a predictor of onchocerciasis elimination. The panels represent 4 examples out of 750 scenarios of precontrol community microfilarial load (CMFL) and history of mass drug administration (MDA). See Figure 1 for a description of how the receiver operating characteristic curves were created. Colored lines represent the Ov16 prevalence measured in different age categories. Symbols represent different threshold values for Ov16 antibody prevalence: 2% (open circle), 5% (open triangle), 10% (open diamond), 25% (solid square), and 50% (solid circle). Ov16 positivity is assumed to be lifelong, but patterns are qualitatively similar when based on the assumption of instant seroreversion. See Web Figure 1 for a complete overview of all 750 scenarios considered and both assumptions about Ov16 seroreversion. A) Precontrol CMFL of 10 microfilariae (mf) per skin snip and 8 rounds of MDA at 60% coverage, resulting in an overall probability of elimination of 82.6%. B) Same scenario as in A, but with 10 instead of 8 MDA rounds, resulting in an overall probability of elimination of 98.4%. C) Precontrol CMFL of 55 mf per skin snip and 13 rounds of MDA at 80% coverage, resulting in an overall probability of elimination of 27.6%. D) Same scenario as in C, but with 15 instead of 13 MDA rounds, resulting in an overall probability of elimination of 70.2%.

Ov16 antibody prevalence in school-age children (ages 5–14 years) provides the best information about the probability of elimination, as illustrated for 4 example scenarios in Figure 2. In younger children (0–4 years), the Ov16 antibody prevalence is generally too close to zero to vary informatively with regards to ongoing transmission. Similarly, the Ov16 antibody prevalence in older children (15–19 years) saturates towards the plateau level of 100% (or the maximum detectable level accounting for imperfect test sensitivity), again resulting in lower variation and thus relatively less information. The age range of 5–14 years performs best throughout the 750 scenarios considered, especially when assuming seroreversion after clearance of infection (Web Figure 1). In general, for scenarios with semiannual MDA, the total amount of information provided by Ov16 serology is lower than for scenarios with annual MDA (threshold sensitivity and specificity are generally lower). This is the result of semiannual MDA resulting in elimination more quickly than annual MDA, providing less time for Ov16 antibody prevalence levels to decline than during annual MDA programs. Related to this, the difference in the amount of information between age groups is lower in scenarios with semiannual MDA.

**Figure 3.**
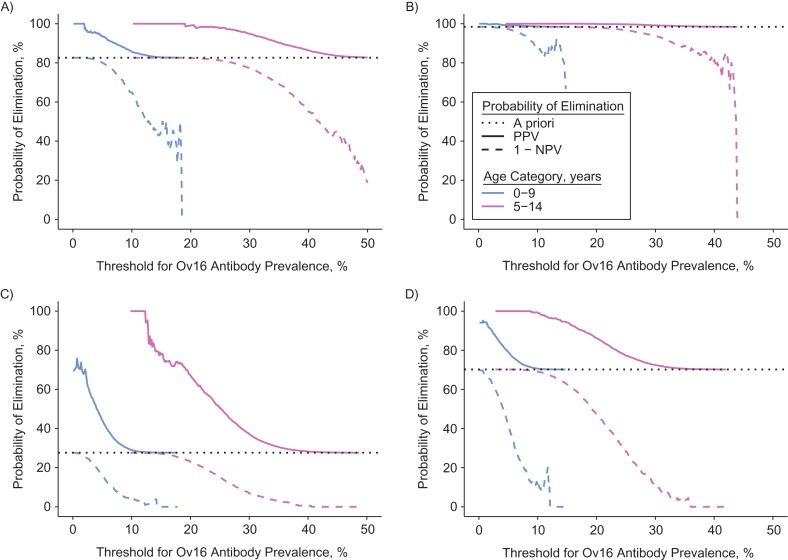
Probability of elimination of onchocerciasis if the Ov16 antibody prevalence is over or under the threshold. The panels represent 4 examples out of 750 scenarios of precontrol CMFL and history of mass drug administration (same selection as in Figure 2). See Figure 1 for a description of how the curves were created. The overall probability of elimination (before Ov16 antibody testing) is represented by the horizontal dotted line in each panel. Here, Ov16 positivity is assumed to be lifelong, but patterns are qualitatively similar when based on the assumption of instant seroreversion. See Web Figure 2 for a complete overview of all 750 scenarios and age groups considered, as well as both assumptions about Ov16 seroreversion. A) Precontrol CMFL of 10 microfilariae (mf) per skin snip and 8 rounds of MDA at 60% coverage. B) Same scenario as in A, but with 10 instead of 8 MDA rounds. C) Precontrol CMFL of 55 mf per skin snip and 13 rounds of MDA at 80% coverage. D) Same scenario as in C, but with 15 instead of 13 MDA rounds.

In Figure 3, we compare the same 4 example scenarios in terms of the probability of elimination if the Ov16 antibody prevalence is under (PPV) or above the threshold (1 minus the negative predictive value), given different choices of diagnostic threshold for Ov16 prevalence (horizontal axis) in children aged either 0–9 or 5–14 years. When sampling children aged 0–9 years, the PPV sometimes cannot reach values close to 100% regardless of the chosen threshold, unless the a priori probability of elimination (before testing, dashed line) is already high. For instance, in Figure 3C and 3D, the maximum PPVs for Ov16 antibody prevalence in children aged 0–9 years are about 75% and 95%, which are associated with threshold values very close to 0%. In contrast, the PPV in children aged 5–14 years can more easily reach 100%, and it does so for Ov16 antibody prevalence threshold values further away from 0%. These patterns are largely consistent throughout the 750 scenarios considered and both hypotheses about the rate of seroreversion (Web Figure 2).

**Figure 4.**
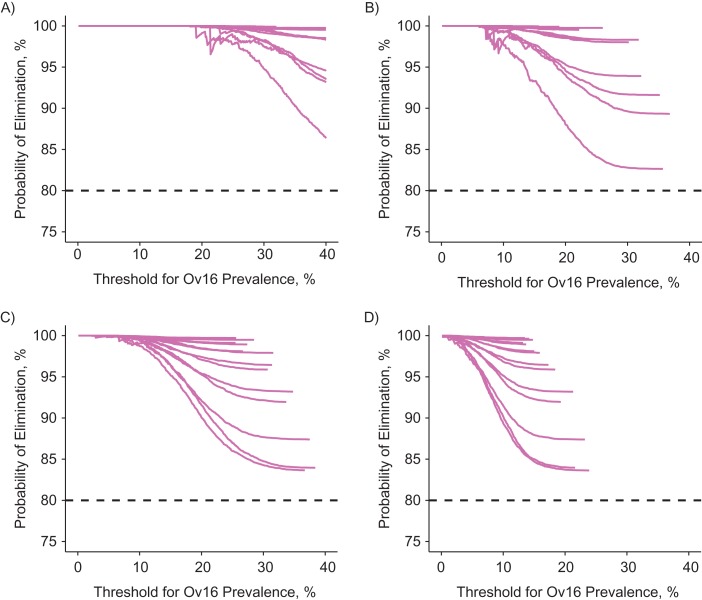
Positive predictive value of Ov16 seroprevalence in children aged 5–14 years for elimination with varying threshold values (horizontal axis). The 4 panels represent 4 combinations of precontrol community microfilarial load (CMFL) and assumptions about reversibility of seropositivity. *Y*-axes represent the probability of elimination if the Ov16 antibody prevalence is under the threshold as defined by the *x*-axis (i.e., the positive predictive value). Lines within each panel represent different histories of mass drug administration (number of rounds and coverage, with annual frequency), but only those that were predicted to result in a priori probability of elimination of at least 80% (i.e., before measuring Ov16 antibody prevalence (dashed line)). Web Figure 3 shows similar plots for all simulated scenarios, sampled age groups, and assumptions about seroreversion. A) Precontrol CMFL of 10 microfilariae (mf) per skin snip with seropositivity assumed to be lifelong. B) Precontrol CMFL as in A, but seropositivity is assumed to revert when the last female worms in a host dies. C) Precontrol CMFL of 55 mf per skin snip with seropositivity assumed to be lifelong. D) Precontrol CMFL as in C, but seropositivity is assumed to revert when the last female worms in a host dies.

In Figure 4, we compare the PPV of different example scenarios with a reasonable a priori probability of elimination (chosen to be >80%)—that is, situations where we actually might consider checking progress towards elimination (see Web Appendix 4 for an overview of the minimum number of MDA rounds required to achieve 80% probability of elimination). For instance, under the assumption of lifelong seropositivity, a PPV of at least 95% can be achieved with an Ov16 threshold of 30% in settings with precontrol community microfilarial load of 10 mf per skin snip, whereas this threshold must be 15% in settings with precontrol community microfilarial load of 55 mf per skin snip. When assuming seroreversion, these threshold values are 13% and 7%, respectively (Figure 4). Threshold values required to achieve PPVs close to 100% are relatively independent of history of control (i.e., lines converge within each panel for low threshold values). Web Figure 3 contains a complete set of curves for all 750 scenarios (i.e., including those with a priori probability of elimination of <80%), sampled age groups, and assumptions about seroreversion. Generally, for settings with semiannual MDA, PPVs close to 100% can be achieved with Ov16 threshold values that are 2–3 percentage points higher than thresholds appropriate for annual MDA.

In Table 2, we compare the required threshold values for Ov16 prevalence in children aged 5–14 years to achieve a PPV of 90%, 95%, or 99% for 5 precontrol infection levels and both hypotheses regarding seroreversion. The most conservative threshold for achieving elimination with 99% probability is on the order of 2.0%, even when accounting for imperfect sensitivity and specificity of the diagnostic test (Web Appendix 2).

**Table 2.** Simulation-Based Threshold Values for Ov16 Antibodies in Children Aged 5–14 Years That Are Consistent With High Probability of Elimination

Precontrol CMFL^a^ (mf/ss)	Threshold for Ov16 Antibody Prevalence Target^b^		
	≥90% Probability of Elimination	≥95% Probability of Elimination	≥99% Probability of Elimination
5	24.5–42.9	19.8–37.3	13.9–28.8
10	18.2–35.3	13.5–29.8	7.8–20.5
30	10.6–24.2	7.6–19.1	2.3–12.6
55	9.5–20.0	6.3–15.3	2.6–9.1
80	8.1–17.8	5.3–13.6	1.9–7.5

Abbreviations: CMFL, community microfilarial load; mf, microfilaria(e); ss, skin snip.

^a^ CMFL is the geometric mean skin microfilarial density in adults of age 20 years or older.

^b^ Threshold values are provided for target probability of elimination (positive predictive value) of 90%, 95%, or 99%. These thresholds were determined for settings where the a priori probability of elimination (before Ov16 testing) is at least 80%. The left value of each reported range is based on the assumption of instant seroreversion; the right value is based on the assumption of no seroreversion (i.e., lifelong seropositivity when it occurs). Threshold values are further based on the assumption that we know the exact serostatus of each individual. In Web Appendix 2 we explain how the reader can interpret our results accounting for misclassification of serostatus.

## DISCUSSION

Our study provides evidence that the predictive value of Ov16 seroprevalence for elimination of onchocerciasis depends heavily on factors that are not or are only partly considered in the current guidelines: precontrol endemicity, MDA strategy, and age group tested. To predict elimination with high certainty (high PPV), threshold values for Ov16 prevalence should be relatively low for more highly endemic settings, annual MDA programs (vs. semiannual), and when sampling younger age groups. We have shown that Ov16 prevalence in school-age children (age 5–14 years) instead of children under age 10 years can provide the best information on prospects of elimination, that a threshold on the order of 2.0% for Ov16 seroprevalence in school-age children is sufficiently conservative to ensure high probability of elimination in the settings where MDA is implemented annually, and that this threshold is even more conservative for settings with semiannual MDA.

The 2.0% threshold is markedly higher than the currently recommended threshold of 0.1%, due to several factors. First, we considered children of ages 5–14 years, among whom seroprevalences are higher than among children aged 0–9 years, but they still decline as a result of MDA (22). If we included only children aged 5–9 years in our sample, which is common practice in field settings, even a hypothetical (and unpractical) threshold of zero percent would not result in 99% probability of elimination. Second, our threshold is per village, while the 0.1% is pooled over a larger area where prevalences might vary from village to village. Our threshold would also need to be considerably lower if applied to a larger area rather than to a village. Third, the threshold can be lower if we correct for the imperfect sensitivity of the Ov16 diagnostic test; for instance, a threshold of 2% true prevalence would translate to a corrected threshold of 1.3% in case of 60% sensitivity and 99.9% specificity of the diagnostic test (see also Web Appendix 2). Last, we assumed that all children were included in the survey; sampling variation is higher when only some of the children are included, meaning that the threshold needs to be lower to achieve the same predicted probability of elimination. Based on the above, we advocate further operational research into the value of different geographical and within-village sampling schemes, as well as the potentially higher value of looking at Ov16 prevalence profiles over wider age ranges rather than summary prevalences over a mix of age groups.

Although the original strategy for Ov16 seroprevalence as an indicator was based on the (implicit) assumption that children born during the MDA program have not been exposed to infection, this assumption is not strictly necessary. With imperfect population coverage of MDA there will always be a chance of residual transmission between MDA rounds. We have shown that even with imperfect coverage of MDA (i.e., 60%–80% in our simulations) one can settle on a single threshold value that works for different MDA histories. Further, in settings where MDA is implemented semiannually, it is very possible that, after 6–7 years of MDA, elimination is achieved but some school-age children are seropositive because of seroconversion before the start of MDA, which is accounted for in our simulations (we explicitly simulate the life histories of individuals). Therefore, the model predicts that in settings where MDA is implemented semiannually, thresholds can be set higher to avoid unnecessary continuation of MDA. However, we chose to present thresholds as applicable for annual MDA programs, because this frequency is most commonly implemented, and note that these thresholds are conservative for settings with semiannual MDA. If MDA is scaled up to a semiannual frequency at a wide scale, further simulations with our model could inform to what extent the threshold could be relaxed.

An operational benefit of targeting school-age children is that this group is easier to sample. Moreover, the relatively higher threshold value requires fewer samples to prove statistically that the prevalence is under the threshold. A study in Uganda highlighted the challenges in meeting the sampling requirements to statistically confirm the 0.1% seropositivity threshold criterion (15). To achieve an upper 95%-confidence bound under a 0.1% threshold, one needs to observe 0 positives among 3,000 independently and identically distributed samples (see Web Appendix 5 for details) (38). In contrast, a threshold of 2% would require zero positives among a mere 148 independently and identically distributed samples. In general, more samples are needed if these are pooled over multiple geographical locations (e.g., villages), because samples from different locations cannot safely be assumed to be independently and identically distributed due to potential differences in Ov16 seroprevalence between locations (the subject of an ongoing modeling study). Still, a higher threshold prevalence means that fewer samples are needed, and that statistically sound inferences might even be made for single locations. Sampling all school-age children in a reasonably large village might provide enough statistical power for inference regarding that particular village.

The decision to stop MDA will most likely be made at the level of implementation units, and therefore pooling results over villages in some form will be inevitable. We strongly advise against calculating Ov16 prevalence simply based on pooled village numerators and denominators, because this might lead to a highly heterogeneous sample with regard to precontrol endemicity, one of the most important determinants of both the prospect of elimination (22, 32) and the predictive value of Ov16 antibody prevalence (as shown in the present study). It would be safer to base stopping criteria on individual village-level prevalences (e.g., a criterion based on the proportion of sampled villages having a prevalence under the threshold value). Pooling Ov16 prevalence over villages that are randomly selected regardless of transmission conditions (as is currently recommended) further increases the risk of diluting Ov16 prevalence levels, requiring a much lower threshold than strictly necessary. We therefore recommend that decision protocols include improved geographical sampling strategies based on trying to select the most highly endemic communities within a transmission focus, and a differentiated approach to evaluating survey results based on precontrol endemicity. In fact, this previously was the strategy for evaluating skin mf prevalence (1). Obviously, in the absence of any reliable precontrol endemicity information, a somewhat more conservative threshold value should be selected.

Our results further underline the importance of the representation of age groups in Ov16 antibody surveys. In our analysis, we assumed that the simulated survey samples were representative of the age and sex distribution of the source population. However, achieving a representative sample might be challenging in reality, and overrepresentation of younger subgroups in the larger sample (e.g., children 5–9 years old, within the proposed target group of school-age children) might lead to deflation of Ov16 seroprevalence and possibly stopping MDA too early. An approach to counter this would be to base decisions on evaluation of Ov16 prevalences in multiple smaller age categories, each associated with an appropriate threshold, although this might not be practical. Therefore, age standardization of Ov16 survey results (22) or even analyzing full age profiles might be warranted.

An important limitation of our study is the lack of understanding of the mechanisms underlying Ov16 seropositivity (i.e., the trigger for Ov16 seroconversion, the longevity of seropositivity, and a potential dependence of the seroreversion rate on age). Of all the potential alternative assumptions regarding the trigger for Ov16 seroconversion (we assumed the first mature worm triggers seroconversion), the most relevant is that seroconversion is triggered exclusively by appearance of mf. If this were the case, threshold values would have to be zero simply because the Ov16 prevalence in children would drop much more quickly than we predict here. Such a low threshold would make it theoretically impossible to evaluate achieving elimination—one cannot statistically prove that a prevalence is 0%. On the other hand, if seroconversion is actually triggered by an earlier life stage than we assume here (e.g., incoming third-stage larvae), thresholds can even be higher, because the Ov16 would drop less quickly during MDA than we currently predict. Most likely, all parasite life stages contribute to some extent to sensitizing the host immune system, especially life stages that live (primarily) outside nodules (i.e., third stage, male adult worms, and mf). As such, we believe that our assumption of the adult worm as the main trigger represents the whole spectrum best. Longitudinal field studies comparing decline in prevalence of skin mf and Ov16 antibodies during MDA might help further elucidate the relative importance of all potential triggers for seroconversion.

To address the issues regarding the longevity of seropositivity, we applied 2 assumptions that are at the polar opposite extremes of the longevity scale (Ov16 seropositivity is either 1) lifelong or 2) lost as soon as the last female worm dies) and based our recommendations on the most conservative one (i.e., resulting in the lowest threshold values). Most likely, the truth lies somewhere in between and will be dependent on multiple factors, including age, frequency of exposure, and duration of exposure. Longitudinal studies would have to be performed to better characterize the potential range of seroreversion responses.

Some uncertainty remains about the efficiency of transmission in populations with very low infection levels. In the model, this efficiency was determined by 2 important assumptions: 1) the level of density dependence in transmission, which is caused by the more efficient transmission of larvae by blackflies at lower infection levels; and 2) the pattern in which human and fly populations mix with each other (we assume homogeneous mixing within a village). If transmission is more efficient in low-prevalence situations than we assumed here—for instance, as a result of highly assortative mixing (i.e., differential mixing of a subset of humans with a subpopulation of blackflies (39))—the threshold should be lower than predicted here. Because it is not precisely known to what extent these 2 factors vary geographically, caution is required and thresholds should be defined conservatively.

In conclusion, our study demonstrates that interpretation of seroprevalence as a measure of ongoing transmission is highly dependent on the age distribution of the sampled population and transmission conditions. The currently recommended strategy to evaluate elimination of onchocerciasis by means of Ov16 antibody prevalence in children under age 10 years in a random selection of villages can be substantially improved. Better predictive power of elimination can be achieved by sampling school-age children (ages 5–14 years), which allows the use of higher threshold values for Ov16 prevalence, and these thresholds can be further increased by taking a differentiated sampling approach based on precontrol endemicity. The operational benefits of these insights are 2-fold: 1) school-age children are easier to sample than the recommended mix of preschool children and school-age children; and 2) much smaller sample sizes are required to statistically confirm that Ov16 prevalence is under the threshold. A challenge remains to prove absence of pockets with residual transmission in a larger area, which requires good geographical coverage of selected survey sites, with purposeful sampling of high-risk settings.

## Supplementary Material

Web Material

## Abbreviations

IgG4: immunoglobulin G4
MDA: mass drug administration
mf: microfilaria(e)
PPV: positive predictive value
