# Supplementary material for: Predictive Value of Ov16 Antibody Prevalence in Different Subpopulations for Elimination of African Onchocerciasis
Source: Am J Epidemiol. 2019 May 7;188(9):1723–32. doi: 10.1093/aje/kwz109 (PMC6735885; doi:10.1093/aje/kwz109)
Supplement: Web Material [file kwz109_coffeng_web_material_final.pdf]

Web Material

Predictive Value of Ov16 Antibody Prevalence in Different Subpopulations for Elimination of African Onchocerciasis

Luc E. Coffeng[§], Wilma A. Stolk[§], Allison Golden, Tala de los Santos, Gonzalo J. Domingo, and Sake J. de Vlas

[§] Authors contributed equally to the work.

Table of contents

WEB APPENDIX 1: THE ONCHOSIM MODEL 1
WEB APPENDIX 2: ACCOUNTING FOR MISCLASSIFICATION OF OV16 SEROSTATUS 2
WEB APPENDIX 3: SIMULATION SCENARIOS 3
WEB APPENDIX 4: MINIMUM NUMBER OF MDA ROUNDS REQUIRED TO ACHIEVE 80% PROBABILITY OF ELIMINATION 4
WEB APPENDIX 5: MINIMUM SAMPLE SIZE REQUIRED GIVEN A THRESHOLD 4
REFERENCES 5
WEB FIGURE 1 7
WEB FIGURE 2 20
WEB FIGURE 3 33

Web Appendix 1: The ONCHOSIM model

ONCHOSIM is an established individual-based model for transmission and control of onchocerciasis (1), and has been used extensively to support decision making in onchocerciasis control programmes in Africa (2–12). ONCHOSIM simulates an African community, the life histories of individual humans and individual worms within humans, and the transmission of infection between humans in the community. The human population changes in composition over time due to birth and death of individuals; random emigration of individuals is assumed to keep the population size more or less constant. Transmission of infection by flies is simulated deterministically, taking account of differences between individuals in the exposure to fly bites (related to age and sex and other random personal factors). Due to this exposure heterogeneity, the rate of acquisition of new infections and resulting infection intensity vary between human individuals, as does their contribution to the infection pool in flies.

The model can simulate the impact of mass drug administration (MDA) and vector control on transmission and infection indicators. The probability that a simulated individual participates in MDA is governed by age and sex (children under five years of age are not treated; a random proportion of women of reproductive age is not treated, assuming that they are pregnant or lactating), and a lifelong compliance factor (the higher the factor, the higher the probability that an individual participates in any given treatment round). We further assume that 5% of the population never participates in MDA (e.g. because of chronic illness or refusal). Under these assumptions the maximum achievable coverage is about 80%.

Predicted trends in infection (Figure 1 in main text, panel A) are obtained by simulating epidemiological surveys in which individuals in the population are skin snipped for detection of microfilariae (mf) at specified moments in time, accounting for the imperfect sensitivity of the skin snip method. All individuals in the population are assumed to participate in the surveys. For reference, Table S1 provides the age and sex distribution of the simulated human population.

A detailed formal description with program code and an overview of parameter quantifications for version 2.58Ap9 of the ONCHOSIM model is provided elsewhere (4). For the current study, we use ONCHOSIM version 2.74, an extended version with new elements to simulate the outcome of antibody detection tests for onchocerciasis infection, as described previously (13).

Table S1. Age and sex distribution of simulated human population.

Age category	Males	Females
0 – 4	9.0%	9.0%
5 – 9	6.9%	6.9%
10 – 14	6.0%	6.0%
15 – 19	5.2%	5.2%
20 – 29	8.3%	8.2%
30 – 49	9.9%	9.8%
50 – 90	4.8%	4.8%

Web Appendix 2: Accounting for misclassification of Ov16 serostatus

Because the risk of misclassification of an individual's serostatus may vary between different POC tests (i.e. *test sensitivity* and *specificity* for detecting antibodies, as opposed to *threshold sensitivity* and *specificity* for predicting elimination), here we perform a reference analysis assuming that we know the exact serostatus of each individual. To account for imperfect test sensitivity and specificity when interpreting the results of our reference analysis, the reader needs to scale the threshold value for "true" Ov16 antibody prevalence, using the standard formula for the association between true and observed prevalence (p_{true} and p_{obs}) given test sensitivity ($Sens_{\text{test}}$) and specificity ($Spec_{\text{test}}$): $p_{\text{obs}} = p_{\text{true}} \cdot Sens_{\text{test}} + (1 - p_{\text{true}}) \cdot (1 - Spec_{\text{test}})$.

For instance, if test sensitivity and specificity are 60% and 99%, a reference threshold value of 2% for “true” Ov16 antibody prevalence (i.e. under the assumption of perfect test sensitivity and specificity) should be interpreted as a threshold value for observed Ov16 prevalence of 2.18% (i.e. $2\% \cdot 60\% + [100\% - 2\%] \times [100\% - 99\%]$). This correction for misclassification does not change the shape of a receiver operator characteristic curve but only the threshold values that it represents. Therefore, we can safely compare the receiver operator characteristic curves for test results from different age groups to see which age groups provide most information regarding whether elimination will be achieved.

Web Appendix 3: Simulation scenarios

For our analysis, we simulated five settings (a village of about 440 individuals) of varying endemicity (annual biting rate of 9409, 10150, 14098, 18078, or 22212, corresponding to pre-control community microfilarial load (CMFL) of about 5, 10, 30, 55, or 80 mf per skin snip) combined with 1 to 25 years of annual or semiannual MDA with ivermectin at one of three levels of MDA coverage (60%, 70% or 80% of the total population), leading to a total of 750 scenarios. Based on previous work (5), treatment was assumed to lead to instantaneous killing of all mf and a temporary complete interruption in the production of mf by all female adult worms. After treatment, mf production recovers gradually over time in all worms, reaching maximum production capacity after 11 months on average (the 2.5^{th} and 97.5^{th} percentile of the variation between worms being 2 and 24 months). Furthermore, ivermectin was assumed to irreversibly reduce adult female worms’ capacity to produce mf after recovery by 35% on average per treatment (2.5^{th} and 97.5^{th} percentiles: 6 and 76%). Effects of multiple treatments were assumed to be cumulative. Both the duration of the recovery period and the irreversible reduction in mf production vary stochastically between worms and treatments. A random 5% of treatments were assumed to be ineffective (e.g. because of malabsorption). Each of the 750 scenarios was simulated 10,000 times, yielding a sample of 10,000 draws from the joint distribution of Ov16 prevalence in various age groups (one year after the last MDA round) and elimination (defined as zero mf prevalence 50 years after the last MDA round) for each scenario (for example, see Figure 1 in main text, panels A-C).

Web Appendix 4: Minimum number of MDA rounds required to achieve 80% probability of elimination

Pre-control community microfilarial load	Mass drug administration coverage	Mass drug administration frequency	
		Annual	Semi-annual
5	60%	6	4
10	60%	8	5
30	60%	17	10
55	60%	25	14
80	60%	>25	18
5	70%	5	3
10	70%	7	4
30	70%	13	9
55	70%	19	12
80	70%	24	15
5	80%	4	3
10	80%	6	4
30	80%	12	8
55	80%	16	11
80	80%	20	14

Web Appendix 5: Minimum sample size required given a threshold

In the discussion section of the main manuscript (fourth paragraph) we explain how many independent and identically distributed samples would be needed to conclude with 95% certainty that the true Ov16 prevalence is under the threshold. Here we describe the statistical approach and assumptions underlying these sample sizes. We assume that the unknown, true Ov16 prevalence follows a beta distribution with shape parameters α and β. From a Bayesian perspective, if one takes a binomial sample of size N and observes x positives and $N - x$ negatives, the posterior distribution of the true (unknown) prevalence is a beta distribution with shape parameters $\alpha = x + 1$ and $\beta = N - x + 1$. For example, if one takes 300 samples and observed zero positives, the estimated true prevalence follows a beta distribution with shape parameters $\alpha = 1$ and $\beta = 301$. The 95th percentile of this distribution is 0.0099 or 0.99%, meaning that the probability that the true prevalence is less than 0.99% is 95%. So, if the target threshold is 2.0%, one would need to observe zero positives among 148 samples to be 95% sure that the prevalence is under 2.0%. This is because the 95th percentile of a beta distribution with $\alpha = 1$ and $\beta = 149$ is 1.9901%, whereas for 147 samples ($\alpha = 1$ and $\beta = 148$) the 95th percentile would be 2.004%.

It is important to highlight that these sample size calculations assume that samples are independently and identically distributed (i.i.d.). If samples are taken from multiple, non-

interchangeable locations (e.g. locations with different transmission settings and/or history of MDA), the method described above underestimates the 95th percentile of the estimated Ov16 prevalence because of correlation between samples from different locations (14). A multi-level model would then be needed to estimate the prevalence and more samples would be needed to achieve the same level of statistical power as for a simple i.i.d. sample. How many more samples would be needed depends on the variation in true prevalence between the sites. A previously suggested approach is assume a standard, conservative “design factor” or “design effect” of 2.0, which is multiplied with the minimum number of samples required under the assumption of i.i.d. observations (14).

References

1. Plaisier AP, van Oortmarssen GJ, Habbema JD, et al. ONCHOSIM: a model and computer simulation program for the transmission and control of onchocerciasis. *Comput Methods Programs Biomed*. 1990;31(1):43–56.
2. Plaisier AP, van Oortmarssen GJ, Remme J, et al. The reproductive lifespan of *Onchocerca volvulus* in West African savanna. *Acta Trop*. 1991;48(4):271–84.
3. Plaisier AP, van Oortmarssen GJ, Remme J, et al. The risk and dynamics of onchocerciasis recrudescence after cessation of vector control. *Bull World Heal. Organ*. 1991;69(2):169–78.
4. Stolk WA, Walker M, Coffeng LE, et al. Required duration of mass ivermectin treatment for onchocerciasis elimination in Africa: a comparative modelling analysis. *Parasit Vectors*. 2015;8(1):552.
5. Plaisier AP, Alley ES, Boatin BA, et al. Irreversible effects of ivermectin on adult parasites in onchocerciasis patients in the Onchocerciasis Control Programme in West Africa. *J Infect Dis*. 1995;172(1):204–10.
6. Plaisier AP, Alley ES, van Oortmarssen GJ, et al. Required duration of combined annual ivermectin treatment and vector control in the Onchocerciasis Control Programme in west Africa. *Bull World Heal. Organ*. 1997;75(3):237–45.
7. Alley WS, van Oortmarssen GJ, Boatin BA, et al. Macrofilaricides and onchocerciasis control, mathematical modelling of the prospects for elimination. *BMC Public Health*. 2001;1:12.
8. Winnen M, Plaisier AP, Alley ES, et al. Can ivermectin mass treatments eliminate onchocerciasis in Africa? *Bull World Heal. Organ*. 2002;80(5):384–91.
9. Coffeng LE, Stolk WA, Zouré HGM, et al. African Programme for Onchocerciasis Control 1995-2015: model-estimated health impact and cost. *PLoS Negl Trop Dis*. 2013;7(1):e2032.
10. Coffeng LE, Stolk WA, Zouré HGM, et al. African Programme for Onchocerciasis Control 1995-2015: updated health impact estimates based on new disability weights. *PLoS Negl Trop Dis*. 2014;8(6):e2759.
11. Coffeng LE, Stolk WA, Hoerauf A, et al. Elimination of African onchocerciasis:

modeling the impact of increasing the frequency of ivermectin mass treatment. *PLoS One*. 2014;9(12):e115886.

12. Kim YE, Remme JHF, Steinmann P, et al. Control, elimination, and eradication of river blindness: scenarios, timelines, and ivermectin treatment needs in Africa. *PLoS Negl Trop Dis*. 2015;9(4):e0003664.
13. Lont YL, Coffeng LE, de Vlas SJ, et al. Modelling Anti-Ov16 IgG4 Antibody Prevalence as an Indicator for Evaluation and Decision Making in Onchocerciasis Elimination Programmes. *PLoS Negl Trop Dis*. 2017;11(1):e0005314.
14. Gass KM. Rethinking the serological threshold for onchocerciasis elimination. *PLoS Negl Trop Dis*. 2018;12(3):e0006249.

Web Figure 1

Receiver operating characteristic curves for Ov16-antibody prevalence as a predictor of onchocerciasis elimination, analogous to Figure 2, but for all 750 simulated scenarios and both assumptions about Ov16 seroreversion.

ROC – Annual mass drug administration

Assumption: seropositivity is lifelong

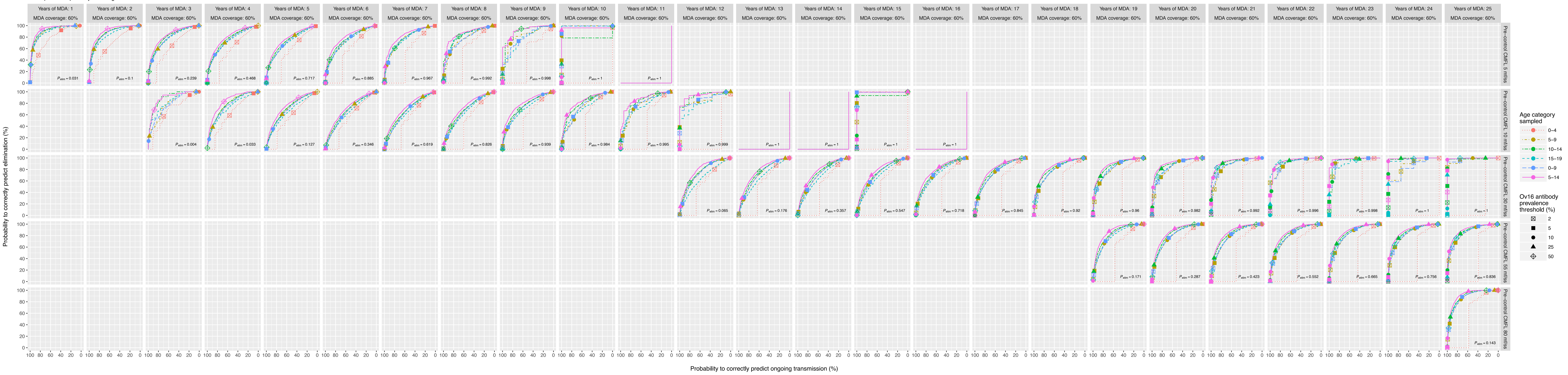

ROC – Annual mass drug administration

Assumption: seropositivity is lifelong

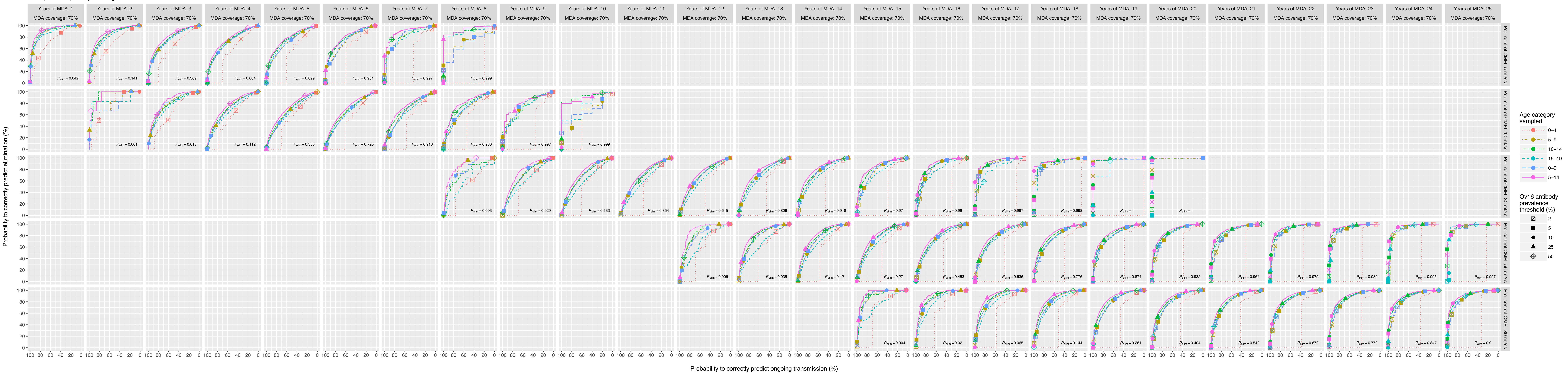

ROC – Annual mass drug administration

Assumption: seropositivity is lifelong

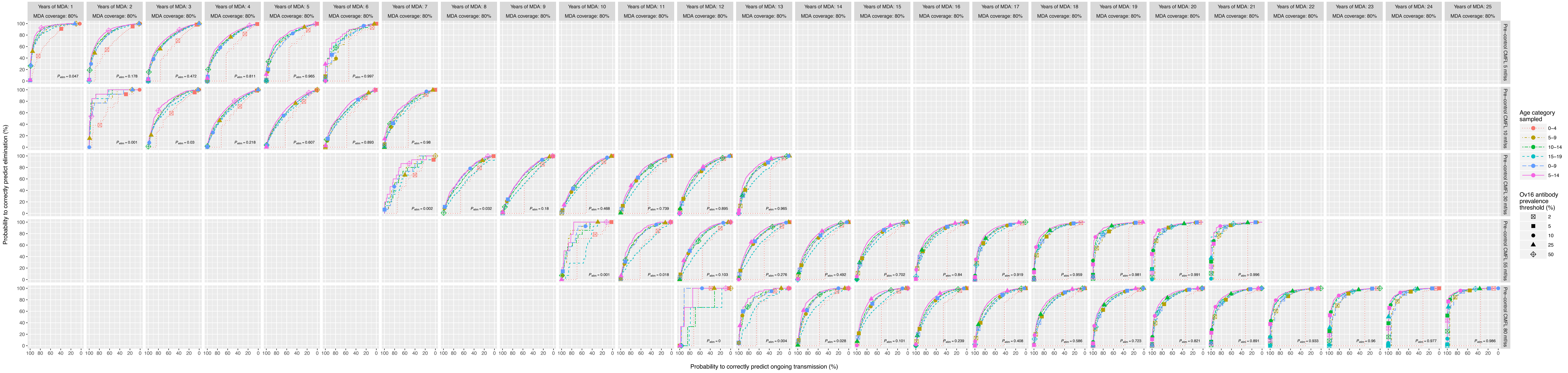

ROC – Annual mass drug administration

Assumption: seropositivity lasts until the last female worm dies

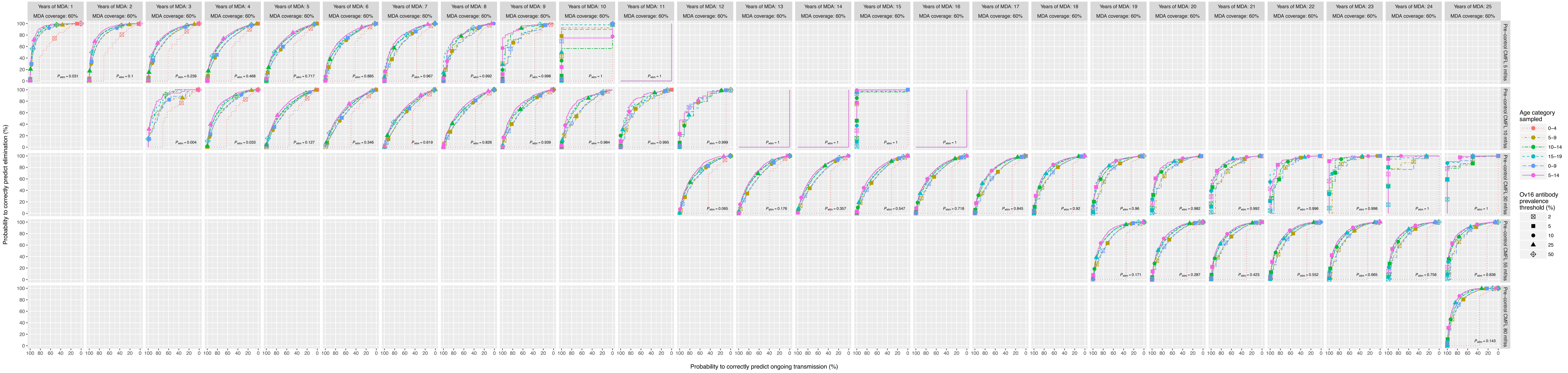

ROC – Annual mass drug administration

Assumption: seropositivity lasts until the last female worm dies

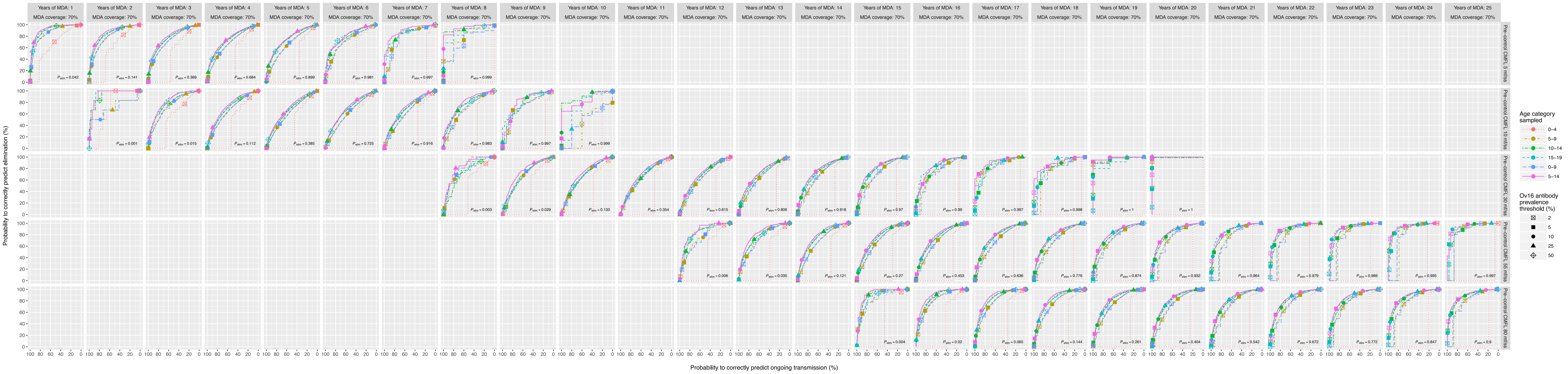

ROC – Annual mass drug administration

Assumption: seropositivity lasts until the last female worm dies

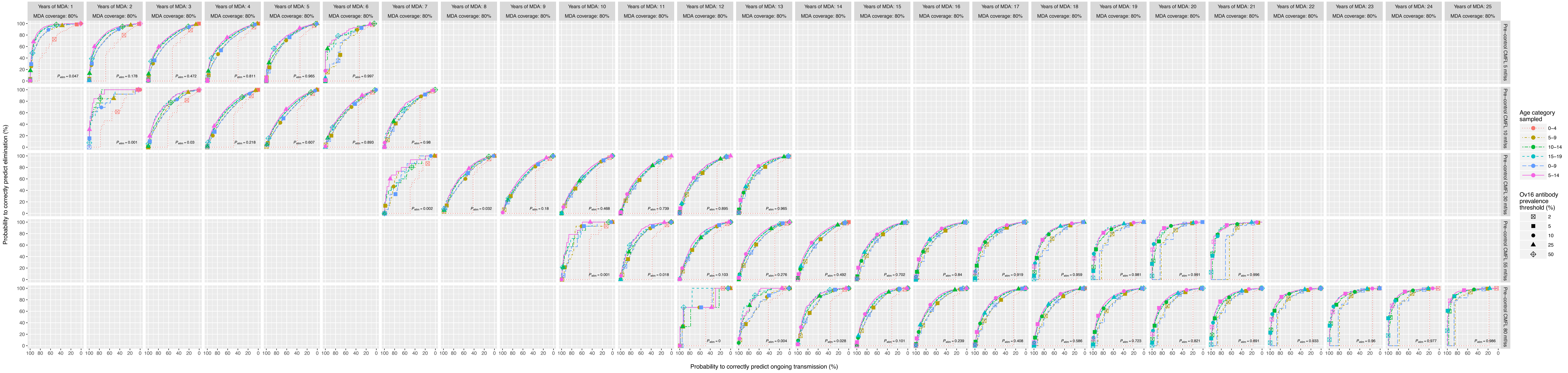

ROC – Semi-annual mass drug administration

Assumption: seropositivity is lifelong

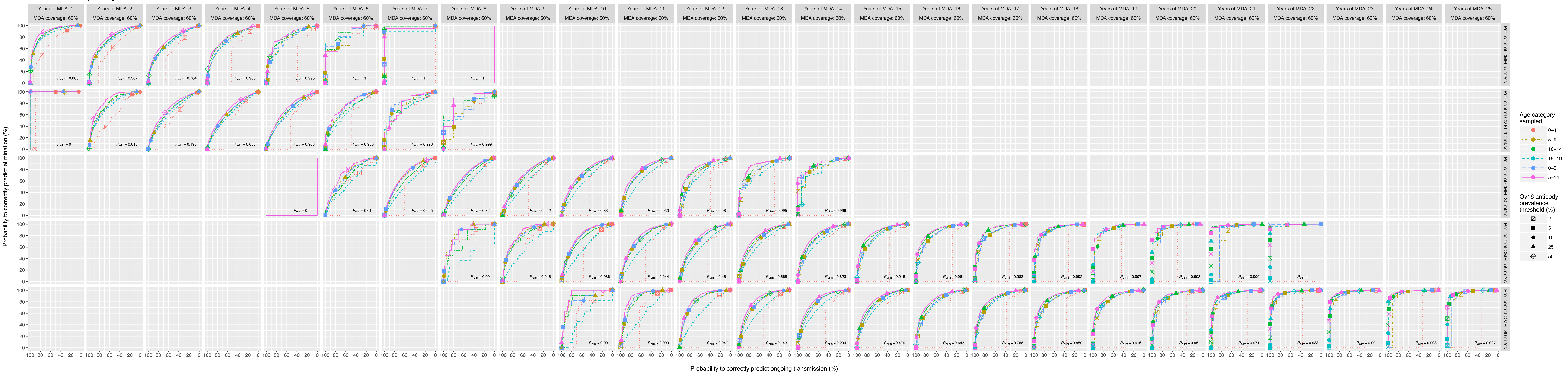

ROC – Semi-annual mass drug administration

Assumption: seropositivity is lifelong

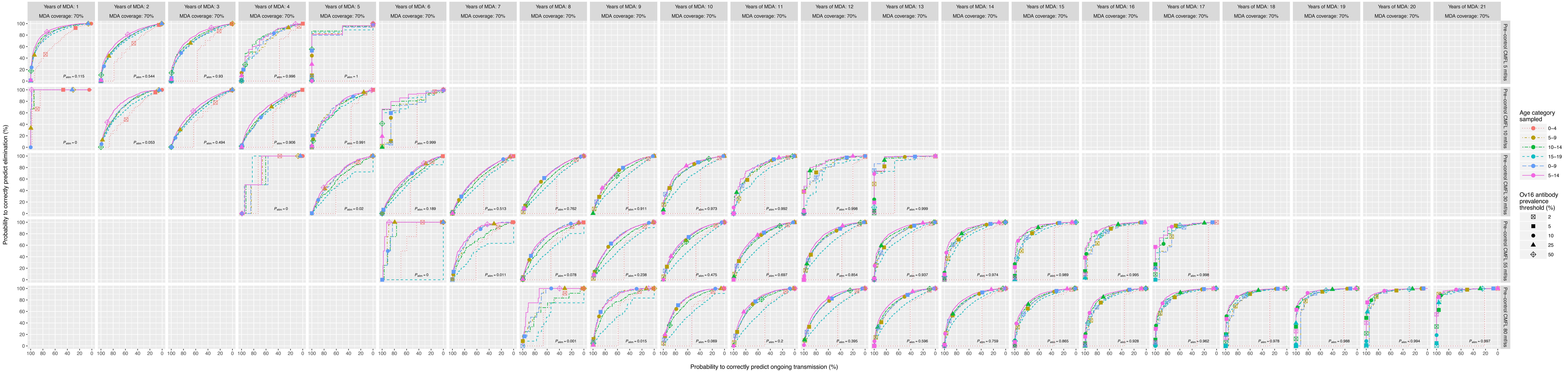

ROC – Semi-annual mass drug administration

Assumption: seropositivity is lifelong

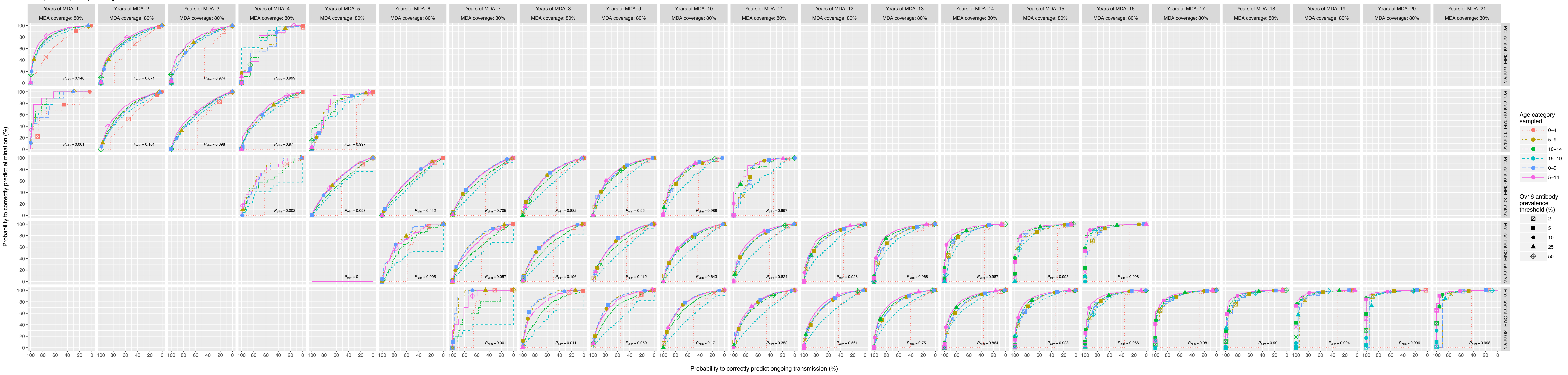

ROC – Semi-annual mass drug administration

Assumption: seropositivity lasts until the last female worm dies

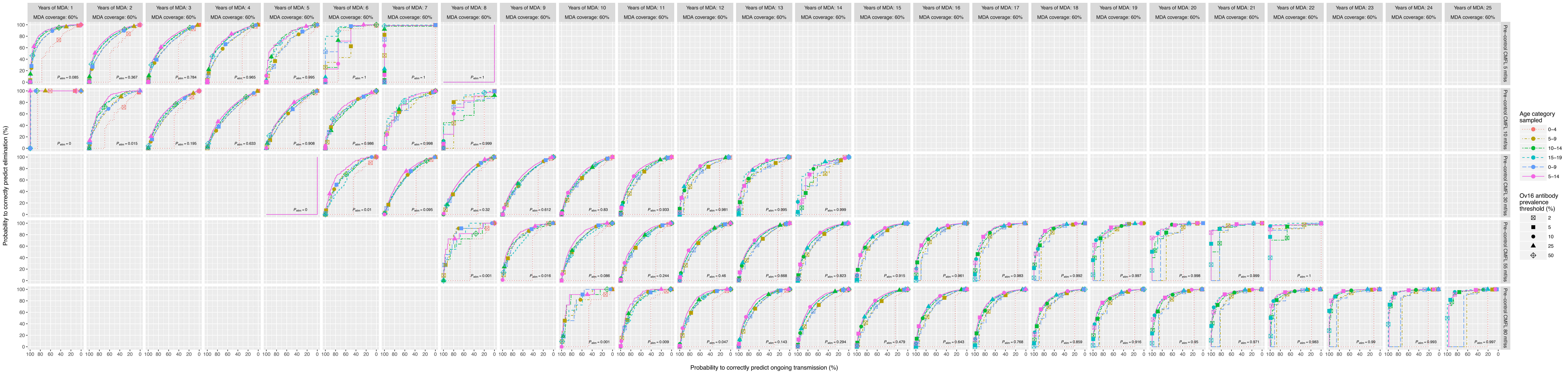

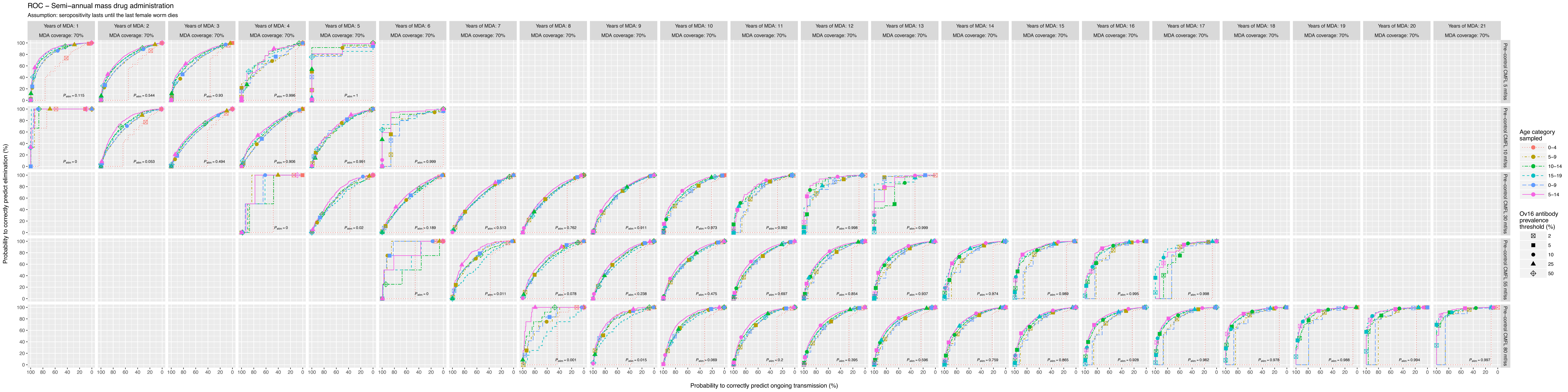

ROC – Semi-annual mass drug administration
Assumption: seropositivity lasts until the last female worm dies
Years of MDA: 1
Years of MDA: 2
Years of MDA: 3
Years of MDA: 4
Years of MDA: 5
Years of MDA: 6
Years of MDA: 7
Years of MDA: 8
Years of MDA: 9
Years of MDA: 10
Years of MDA: 11
Years of MDA: 12
Years of MDA: 13
Years of MDA: 14
Years of MDA: 15
Years of MDA: 16
Years of MDA: 17
Years of MDA: 18
Years of MDA: 19
Years of MDA: 20
Years of MDA: 21
MDA coverage: 70%
Pre-control CMFL 5 mf/ss
Pre-control CMFL 10 mf/ss
Pre-control CMFL 30 mf/ss
Pre-control CMFL 55 mf/ss
Pre-control CMFL 80 mf/ss
P_{elim} = 0.115
P_{elim} = 0.544
P_{elim} = 0.93
P_{elim} = 0.996
P_{elim} = 1
P_{elim} = 0
P_{elim} = 0.053
P_{elim} = 0.494
P_{elim} = 0.906
P_{elim} = 0.991
P_{elim} = 0.999
P_{elim} = 0
P_{elim} = 0.02
P_{elim} = 0.189
P_{elim} = 0.513
P_{elim} = 0.762
P_{elim} = 0.911
P_{elim} = 0.973
P_{elim} = 0.992
P_{elim} = 0.998
P_{elim} = 0.999
P_{elim} = 0
P_{elim} = 0.011
P_{elim} = 0.078
P_{elim} = 0.238
P_{elim} = 0.475
P_{elim} = 0.697
P_{elim} = 0.854
P_{elim} = 0.937
P_{elim} = 0.974
P_{elim} = 0.989
P_{elim} = 0.995
P_{elim} = 0.998
P_{elim} = 0.001
P_{elim} = 0.015
P_{elim} = 0.069
P_{elim} = 0.2
P_{elim} = 0.395
P_{elim} = 0.596
P_{elim} = 0.759
P_{elim} = 0.865
P_{elim} = 0.928
P_{elim} = 0.962
P_{elim} = 0.978
P_{elim} = 0.988
P_{elim} = 0.994
P_{elim} = 0.997
Probability to correctly predict elimination (%)
Probability to correctly predict ongoing transmission (%)
Age category sampled
0–4
5–9
10–14
15–19
0–9
5–14
Ov16 antibody prevalence threshold (%)
2
5
10
25
50

ROC – Semi-annual mass drug administration

Assumption: seropositivity lasts until the last female worm dies

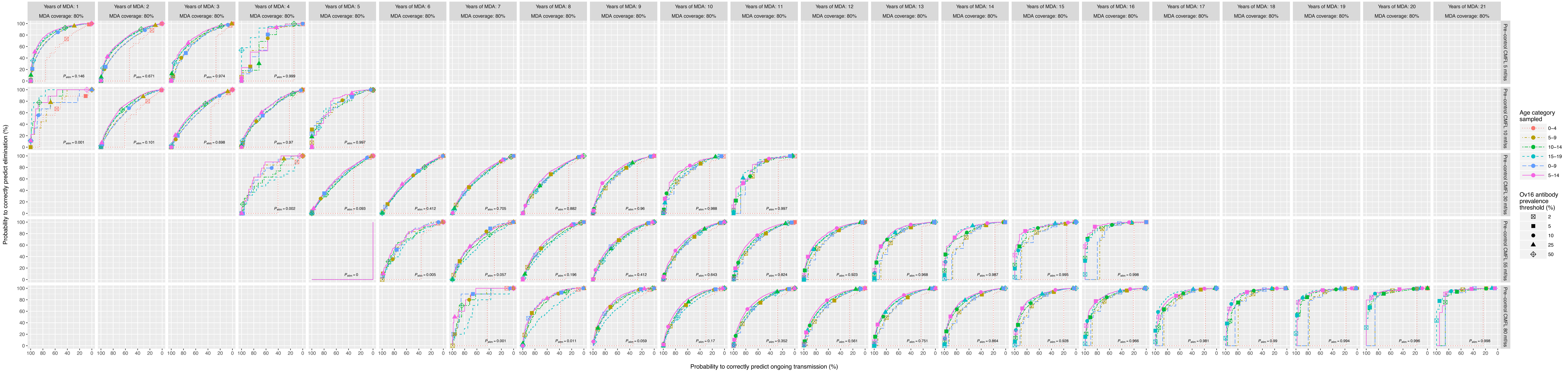

Web Figure 2

Positive and negative predictive value of Ov16-antibody prevalence for varying threshold values and age groups, analogous to Figure 3, but for all 750 simulated scenarios and both assumptions about Ov16 seroreversion.

Predictive Value – Annual Mass Drug Administration

Assumption: Seropositivity is Lifelong

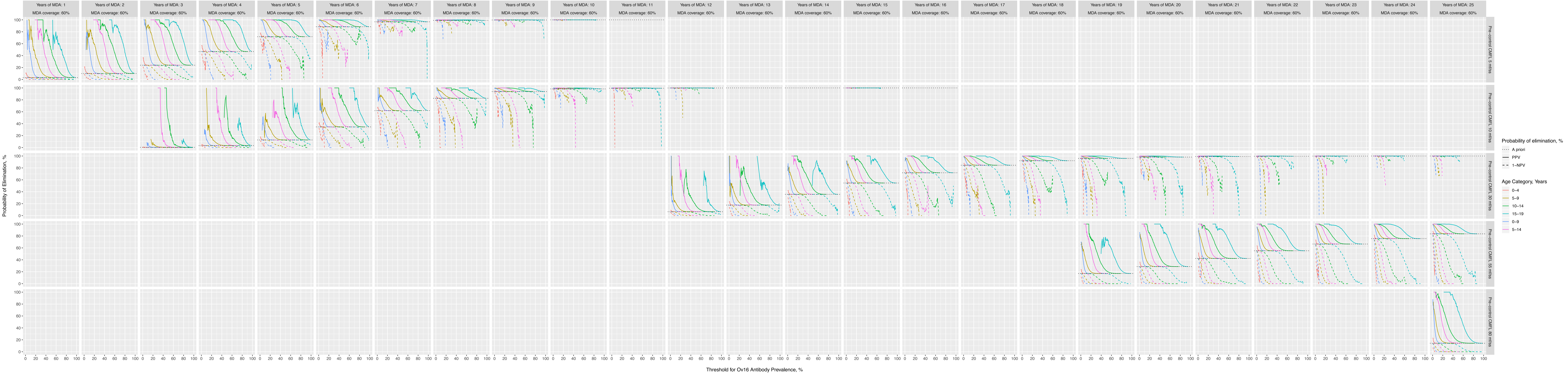

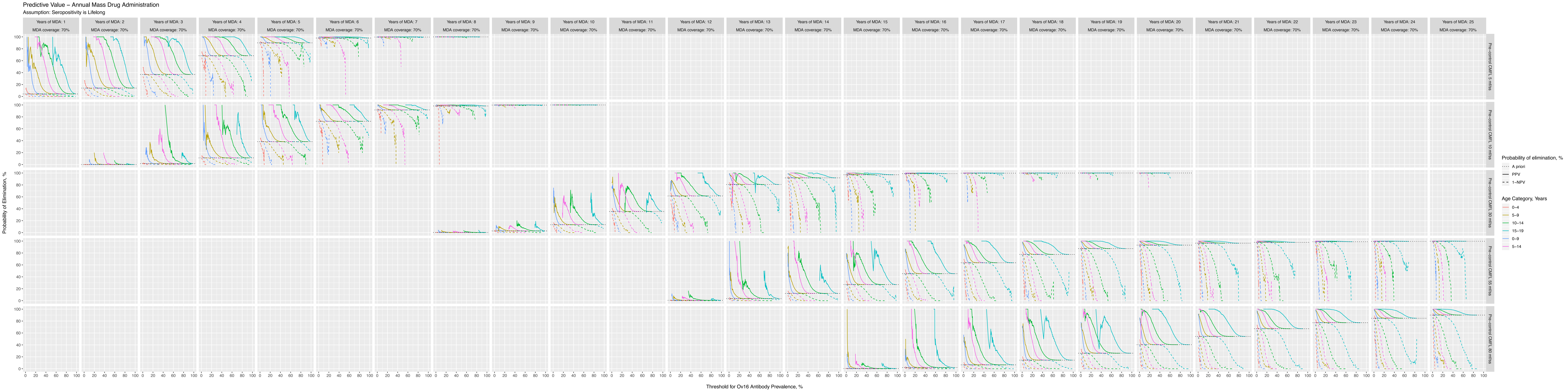

Predictive Value – Annual Mass Drug Administration
Assumption: Seropositivity is Lifelong
Years of MDA: 1
Years of MDA: 2
Years of MDA: 3
Years of MDA: 4
Years of MDA: 5
Years of MDA: 6
Years of MDA: 7
Years of MDA: 8
Years of MDA: 9
Years of MDA: 10
Years of MDA: 11
Years of MDA: 12
Years of MDA: 13
Years of MDA: 14
Years of MDA: 15
Years of MDA: 16
Years of MDA: 17
Years of MDA: 18
Years of MDA: 19
Years of MDA: 20
Years of MDA: 21
Years of MDA: 22
Years of MDA: 23
Years of MDA: 24
Years of MDA: 25
MDA coverage: 70%
Pre-control CMFL 5 mf/ss
Pre-control CMFL 10 mf/ss
Pre-control CMFL 30 mf/ss
Pre-control CMFL 55 mf/ss
Pre-control CMFL 80 mf/ss
Probability of Elimination, %
Threshold for Ov16 Antibody Prevalence, %
Probability of elimination, %
A priori
PPV
1–NPV
Age Category, Years
0–4
5–9
10–14
15–19
0–9
5–14

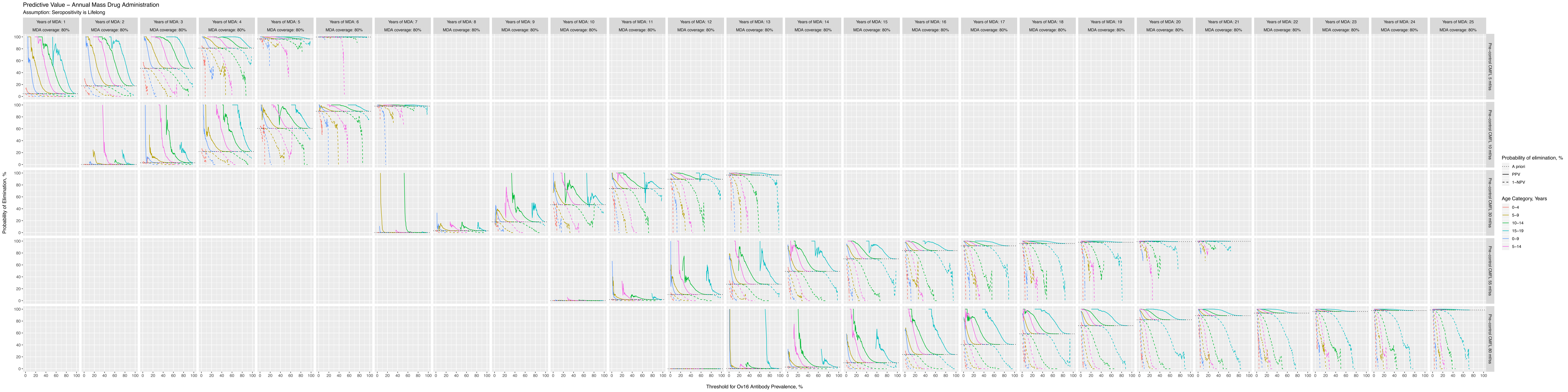

Predictive Value – Annual Mass Drug Administration
Assumption: Seropositivity is Lifelong
Years of MDA: 1
Years of MDA: 2
Years of MDA: 3
Years of MDA: 4
Years of MDA: 5
Years of MDA: 6
Years of MDA: 7
Years of MDA: 8
Years of MDA: 9
Years of MDA: 10
Years of MDA: 11
Years of MDA: 12
Years of MDA: 13
Years of MDA: 14
Years of MDA: 15
Years of MDA: 16
Years of MDA: 17
Years of MDA: 18
Years of MDA: 19
Years of MDA: 20
Years of MDA: 21
Years of MDA: 22
Years of MDA: 23
Years of MDA: 24
Years of MDA: 25
MDA coverage: 80%
Pre–control CMFL 5 mf/ss
Pre–control CMFL 10 mf/ss
Pre–control CMFL 30 mf/ss
Pre–control CMFL 55 mf/ss
Pre–control CMFL 80 mf/ss
Probability of Elimination, %
Threshold for Ov16 Antibody Prevalence, %
Probability of elimination, %
A priori
PPV
1–NPV
Age Category, Years
0–4
5–9
10–14
15–19
0–9
5–14

Predictive Value – Annual Mass Drug Administration

Assumption: Seropositivity Lasts Until the Last Female Worm Dies

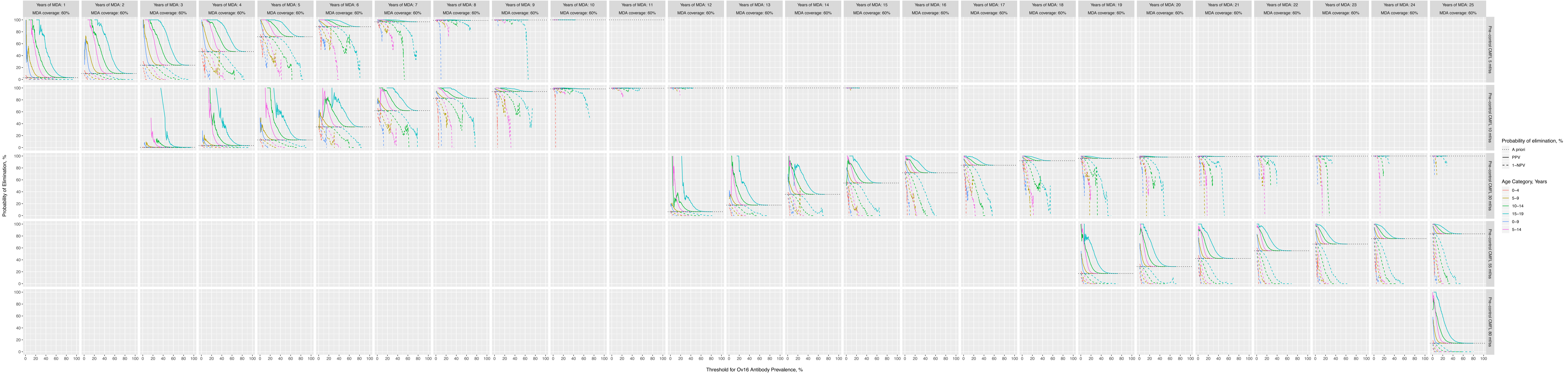

Predictive Value – Annual Mass Drug Administration
Assumption: Seropositivity Lasts Until the Last Female Worm Dies

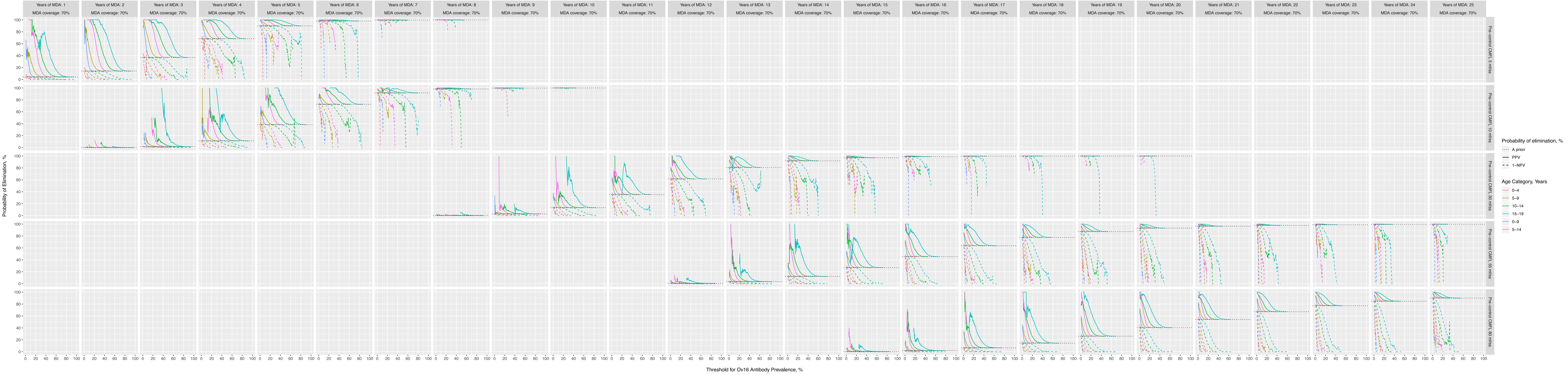

Predictive Value – Annual Mass Drug Administration

Assumption: Seropositivity Lasts Until the Last Female Worm Dies

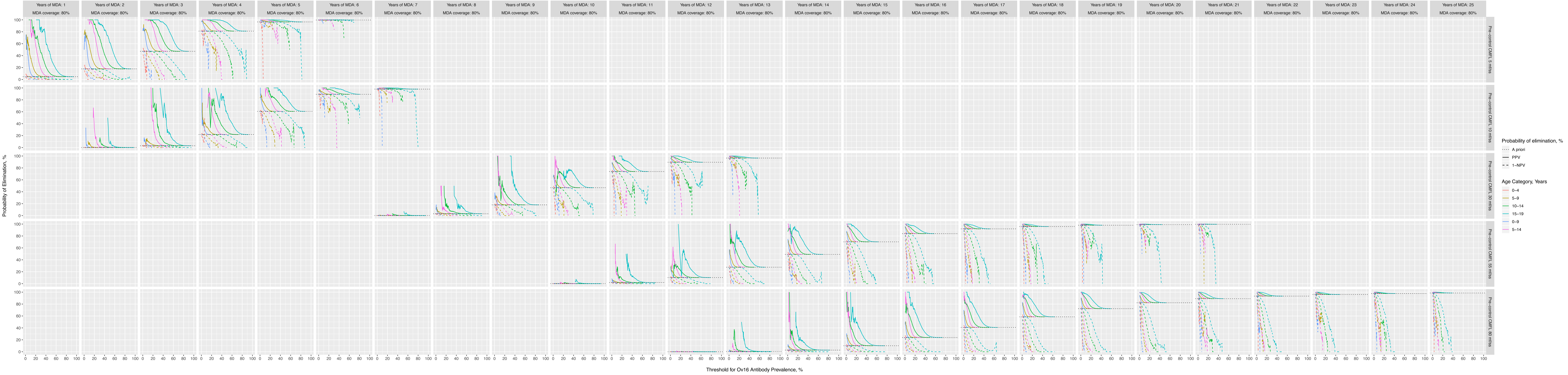

Predictive Value – Semi–Annual Mass Drug Administration

Assumption: Seropositivity is Lifelong

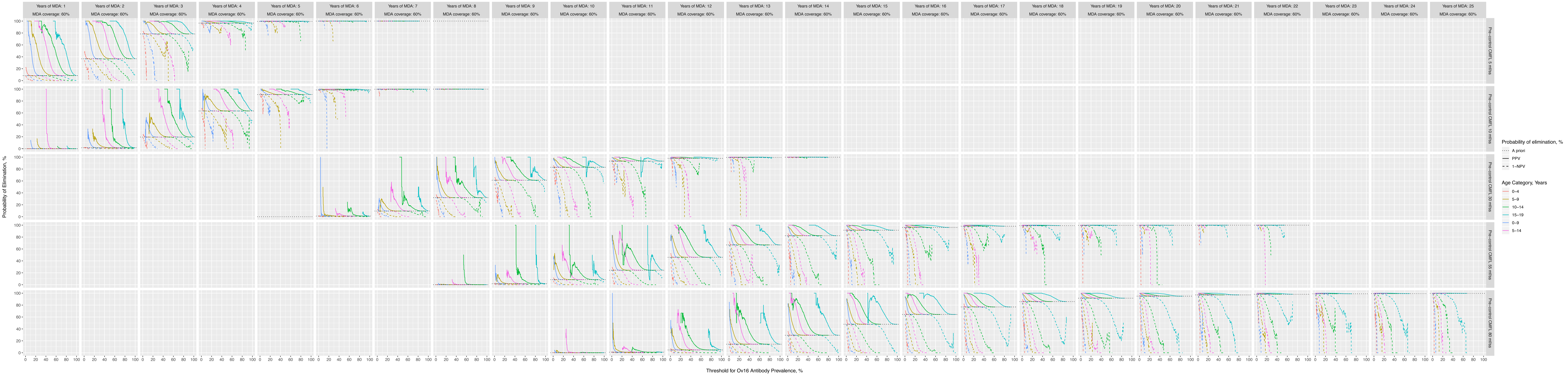

Predictive Value – Semi–Annual Mass Drug Administration
Assumption: Seropositivity is Lifelong

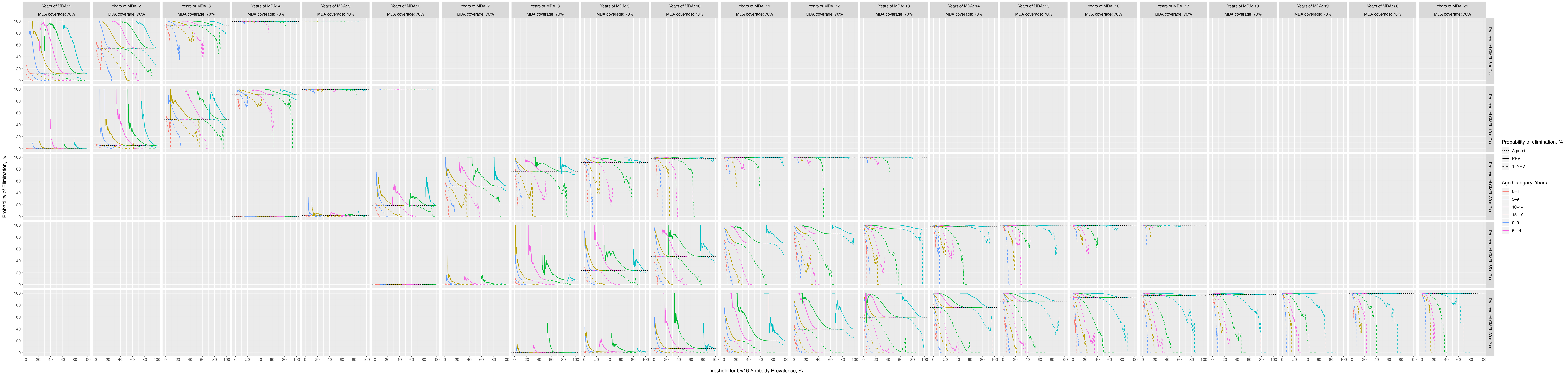

Predictive Value – Semi–Annual Mass Drug Administration

Assumption: Seropositivity is Lifelong

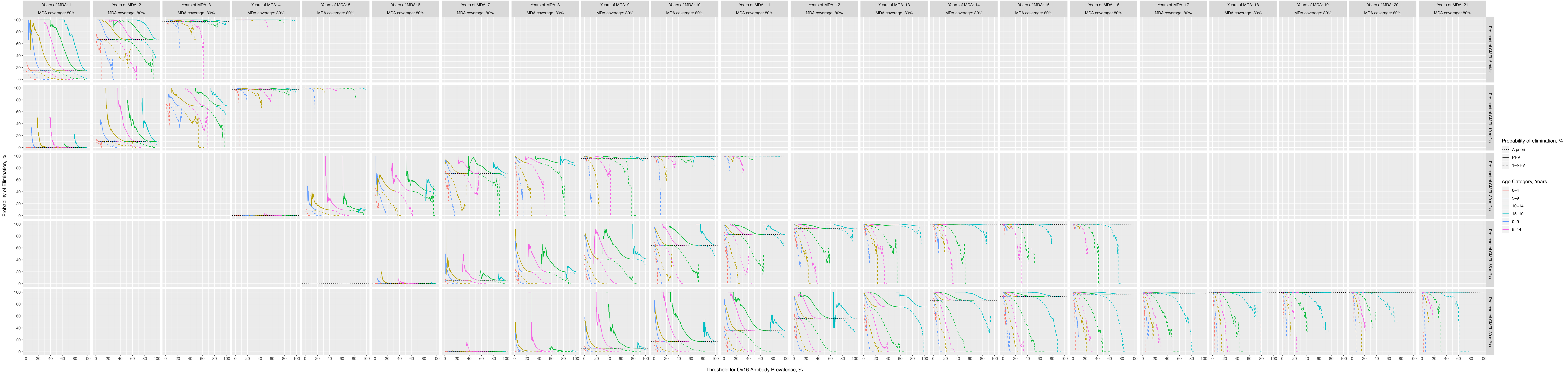

Predictive Value – Semi–Annual Mass Drug Administration

Assumption: Seropositivity Lasts Until the Last Female Worm Dies

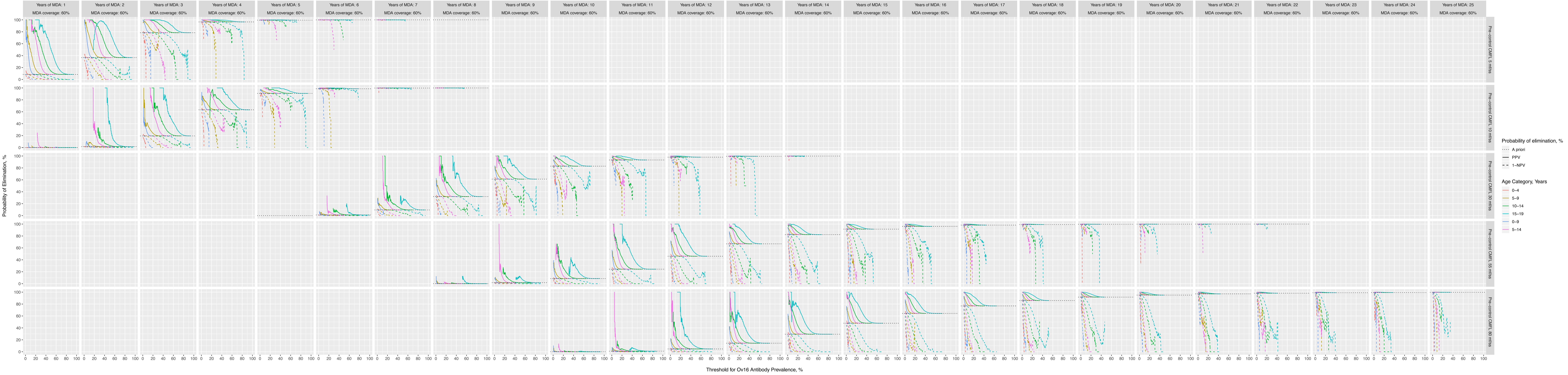

Predictive Value – Semi–Annual Mass Drug Administration

Assumption: Seropositivity Lasts Until the Last Female Worm Dies

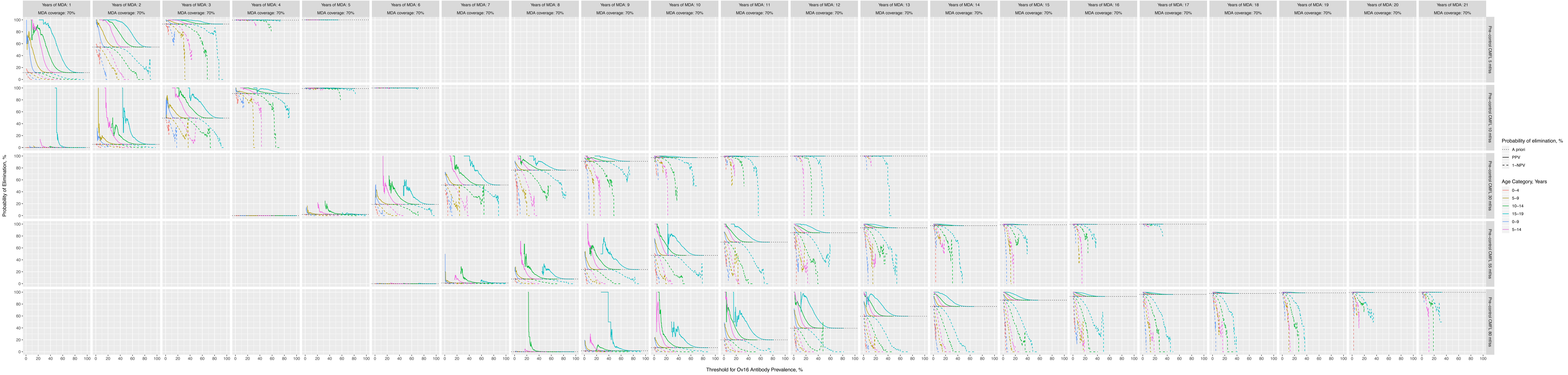

Predictive Value – Semi–Annual Mass Drug Administration

Assumption: Seropositivity Lasts Until the Last Female Worm Dies

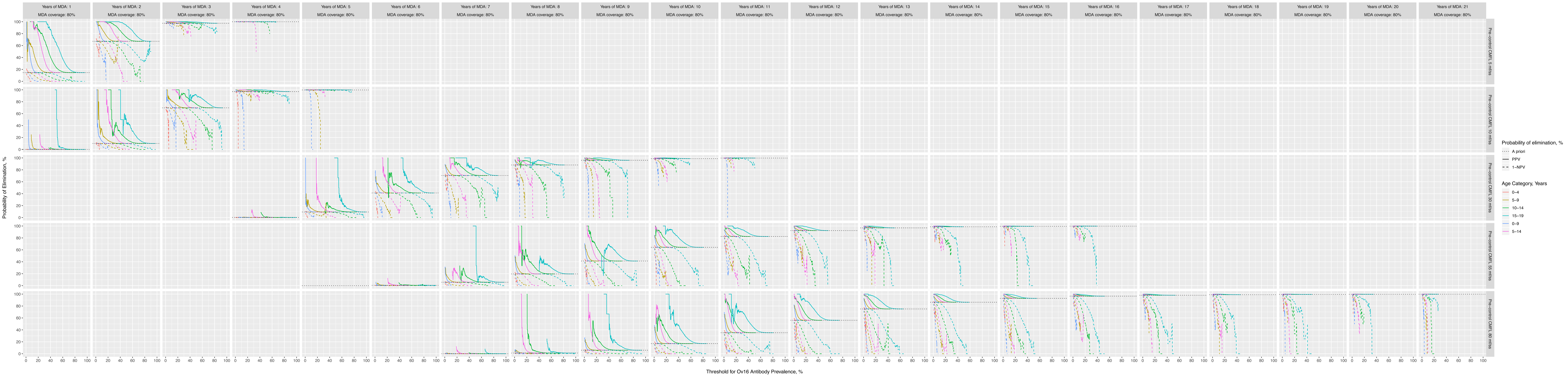

Web Figure 3

Positive predictive value for elimination of varying Ov16-antibody prevalence thresholds in different sampled populations, analogous to Figure 4, but for all 750 simulated scenarios and both assumptions about Ov16 seroreversion, and the subsets thereof that result in an overall probability of elimination of at least 60% or 80%.

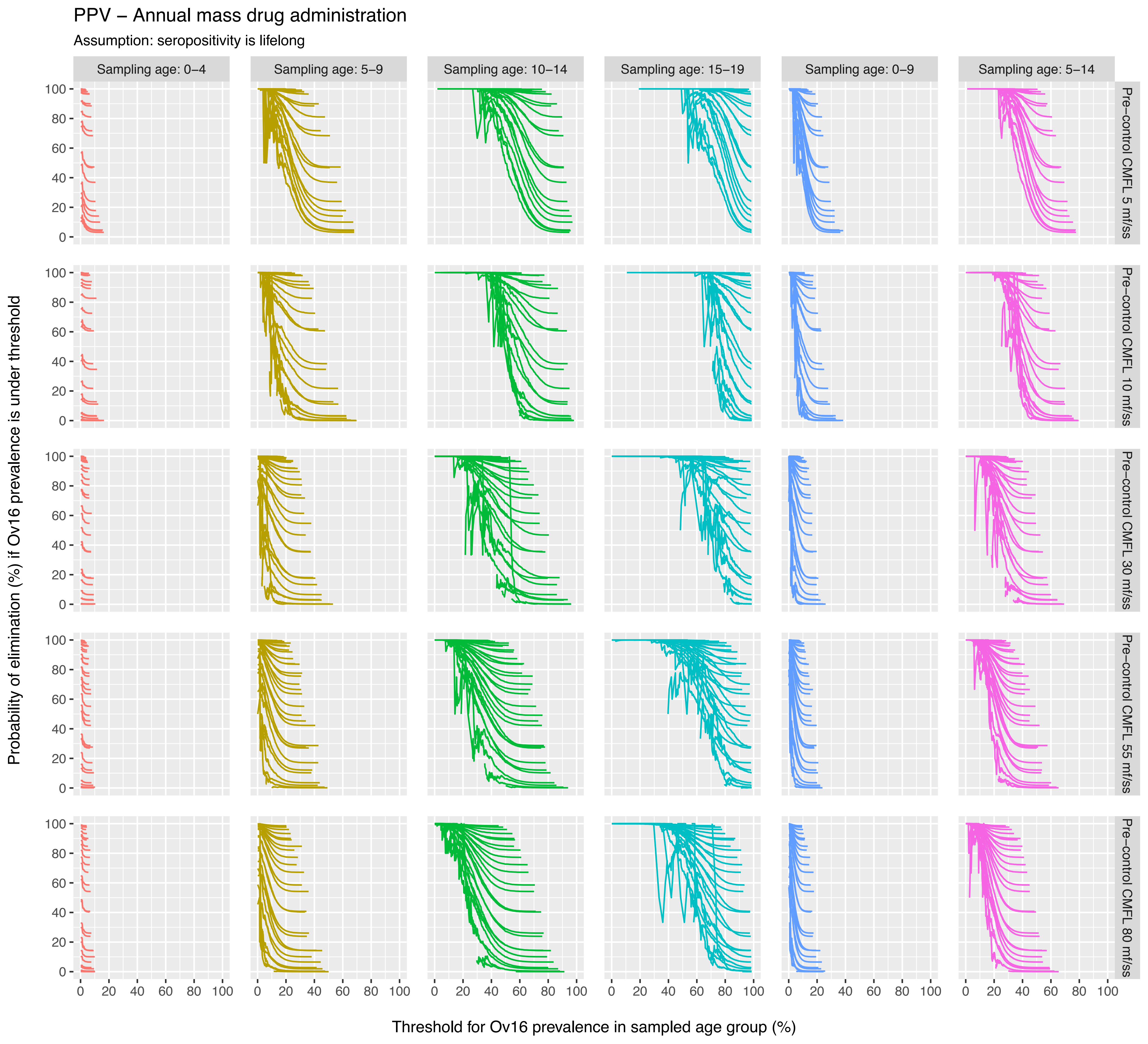
PPV – Annual mass drug administration
Assumption: seropositivity is lifelong
Sampling age: 0–4
Sampling age: 5–9
Sampling age: 10–14
Sampling age: 15–19
Sampling age: 0–9
Sampling age: 5–14
Pre–control CMFL 5 mf/ss
Pre–control CMFL 10 mf/ss
Pre–control CMFL 30 mf/ss
Pre–control CMFL 55 mf/ss
Pre–control CMFL 80 mf/ss
Probability of elimination (%) if Ov16 prevalence is under threshold
Threshold for Ov16 prevalence in sampled age group (%)
0
20
40
60
80
100

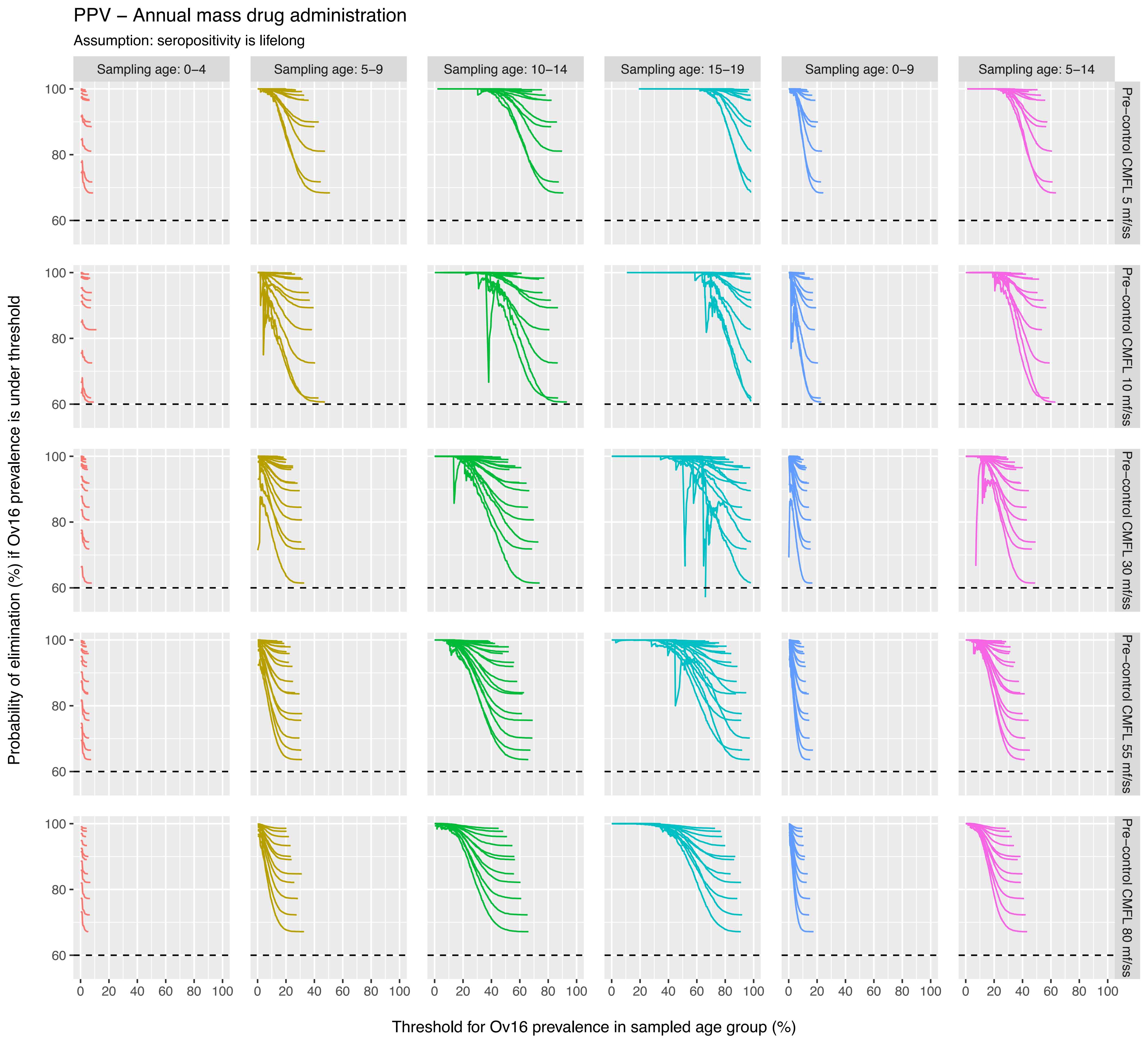
PPV – Annual mass drug administration
Assumption: seropositivity is lifelong
Sampling age: 0–4
Sampling age: 5–9
Sampling age: 10–14
Sampling age: 15–19
Sampling age: 0–9
Sampling age: 5–14
Pre–control CMFL 5 mf/ss
Pre–control CMFL 10 mf/ss
Pre–control CMFL 30 mf/ss
Pre–control CMFL 55 mf/ss
Pre–control CMFL 80 mf/ss
Probability of elimination (%) if Ov16 prevalence is under threshold
Threshold for Ov16 prevalence in sampled age group (%)
100
80
60
0
20
40
60
80
100

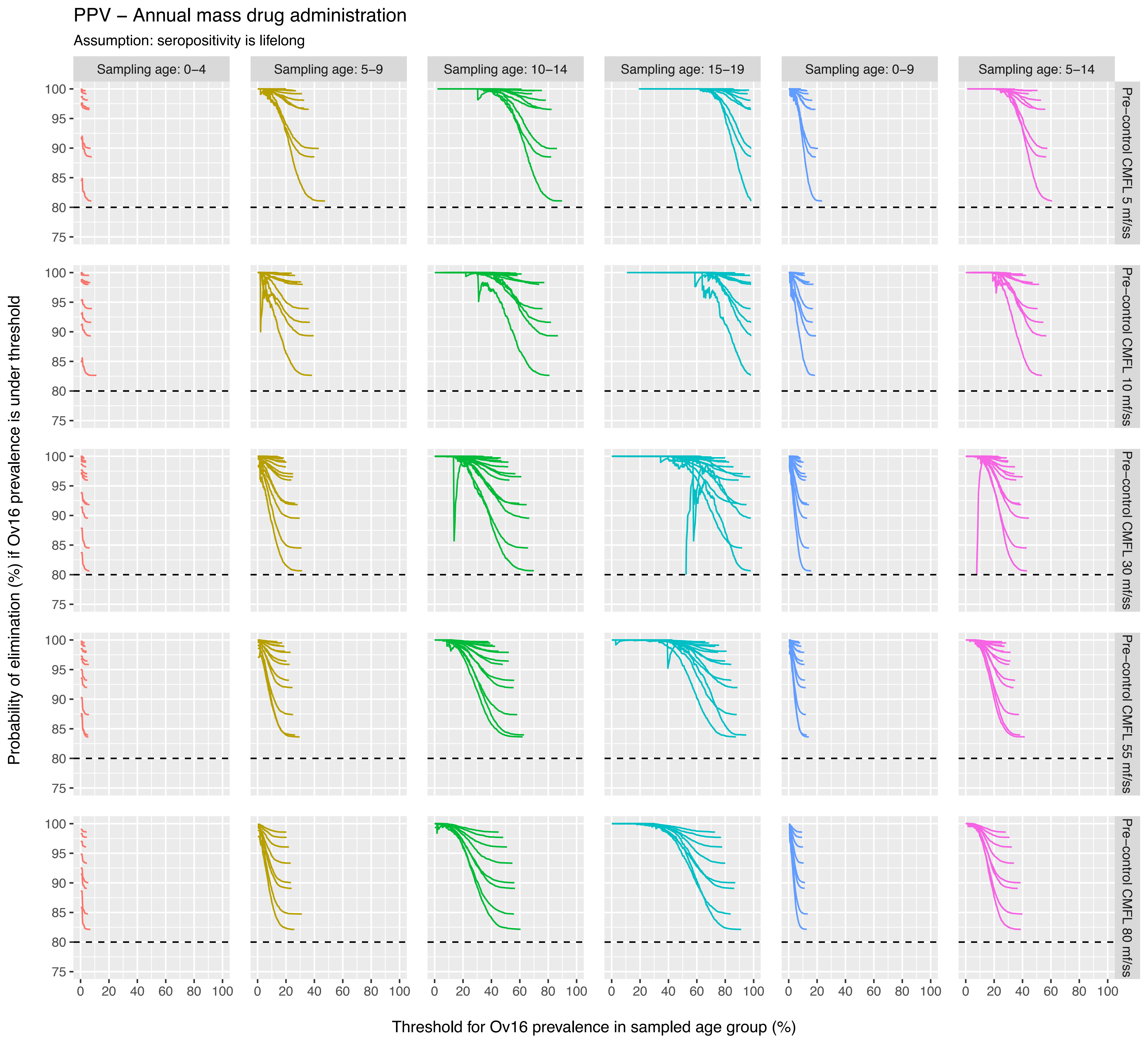
PPV – Annual mass drug administration
Assumption: seropositivity is lifelong
Sampling age: 0–4
Sampling age: 5–9
Sampling age: 10–14
Sampling age: 15–19
Sampling age: 0–9
Sampling age: 5–14
Pre–control CMFL 5 mf/ss
Pre–control CMFL 10 mf/ss
Pre–control CMFL 30 mf/ss
Pre–control CMFL 55 mf/ss
Pre–control CMFL 80 mf/ss
Probability of elimination (%) if Ov16 prevalence is under threshold
Threshold for Ov16 prevalence in sampled age group (%)
75
80
85
90
95
100
0
20
40
60
80
100

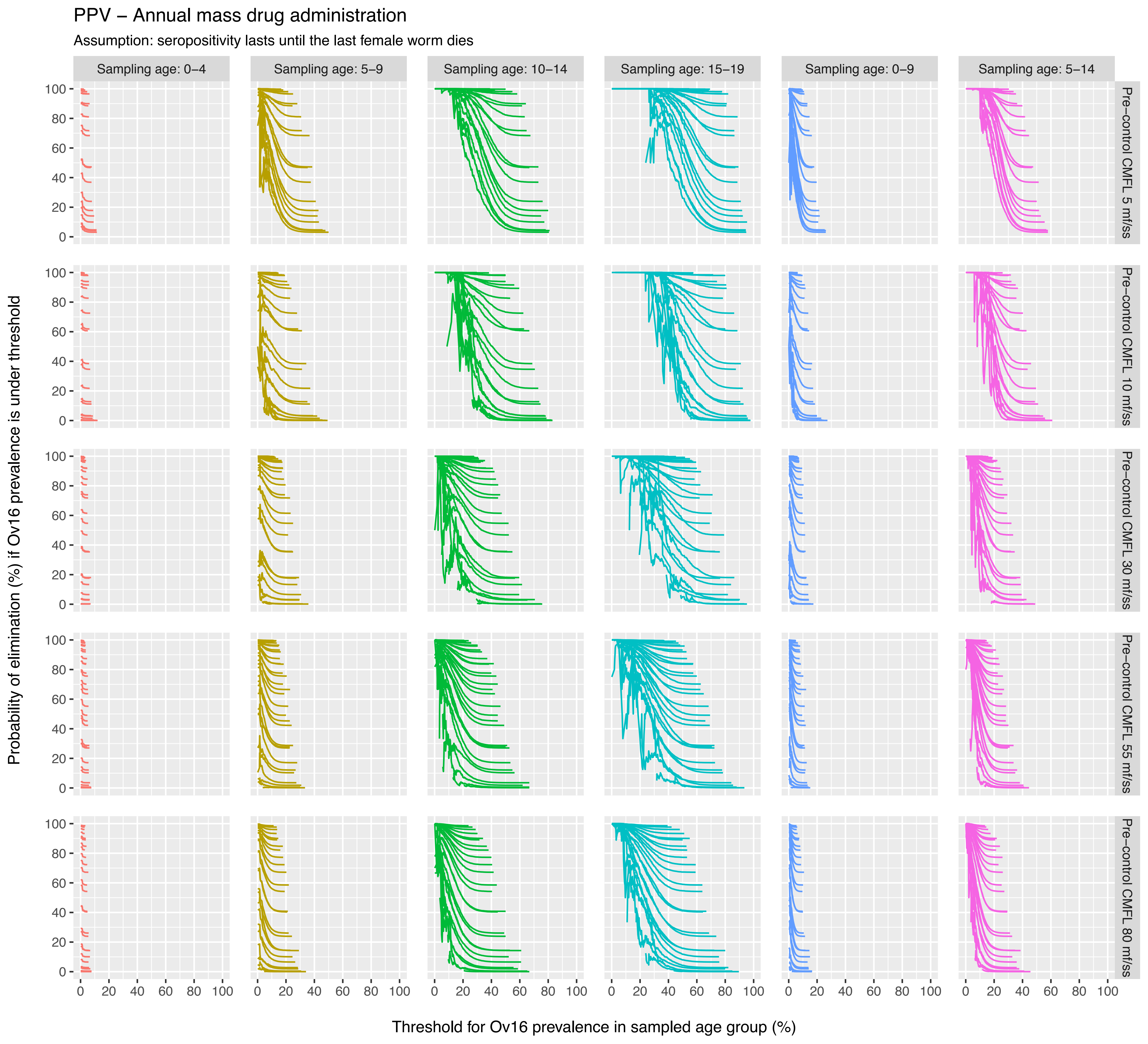
PPV – Annual mass drug administration
Assumption: seropositivity lasts until the last female worm dies
Sampling age: 0–4
Sampling age: 5–9
Sampling age: 10–14
Sampling age: 15–19
Sampling age: 0–9
Sampling age: 5–14
Pre–control CMFL 5 mf/ss
Pre–control CMFL 10 mf/ss
Pre–control CMFL 30 mf/ss
Pre–control CMFL 55 mf/ss
Pre–control CMFL 80 mf/ss
Probability of elimination (%) if Ov16 prevalence is under threshold
Threshold for Ov16 prevalence in sampled age group (%)
0
20
40
60
80
100

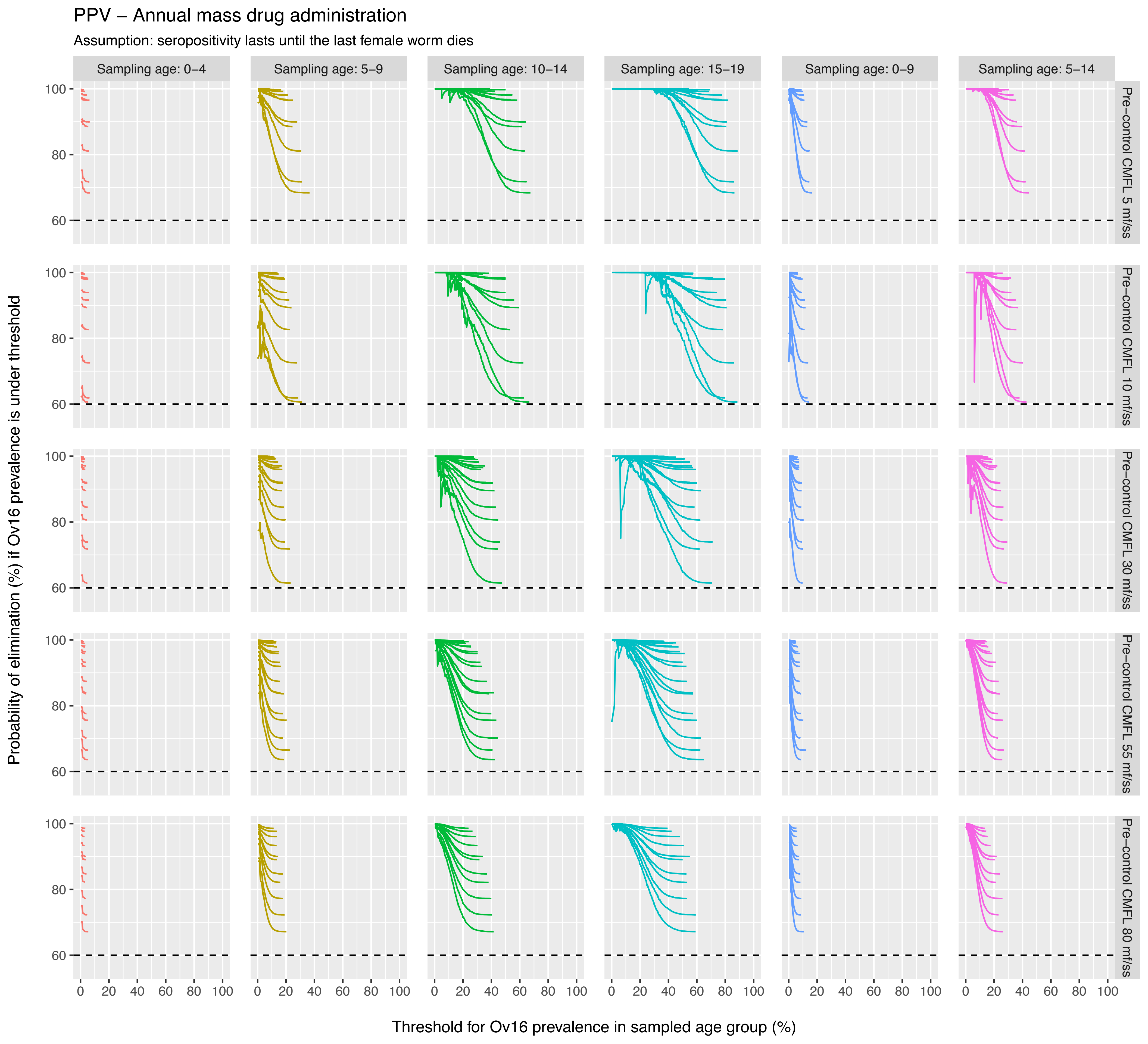
PPV – Annual mass drug administration
Assumption: seropositivity lasts until the last female worm dies
Sampling age: 0–4
Sampling age: 5–9
Sampling age: 10–14
Sampling age: 15–19
Sampling age: 0–9
Sampling age: 5–14
Pre–control CMFL 5 mf/ss
Pre–control CMFL 10 mf/ss
Pre–control CMFL 30 mf/ss
Pre–control CMFL 55 mf/ss
Pre–control CMFL 80 mf/ss
Probability of elimination (%) if Ov16 prevalence is under threshold
Threshold for Ov16 prevalence in sampled age group (%)
100
80
60
0
20
40
60
80
100

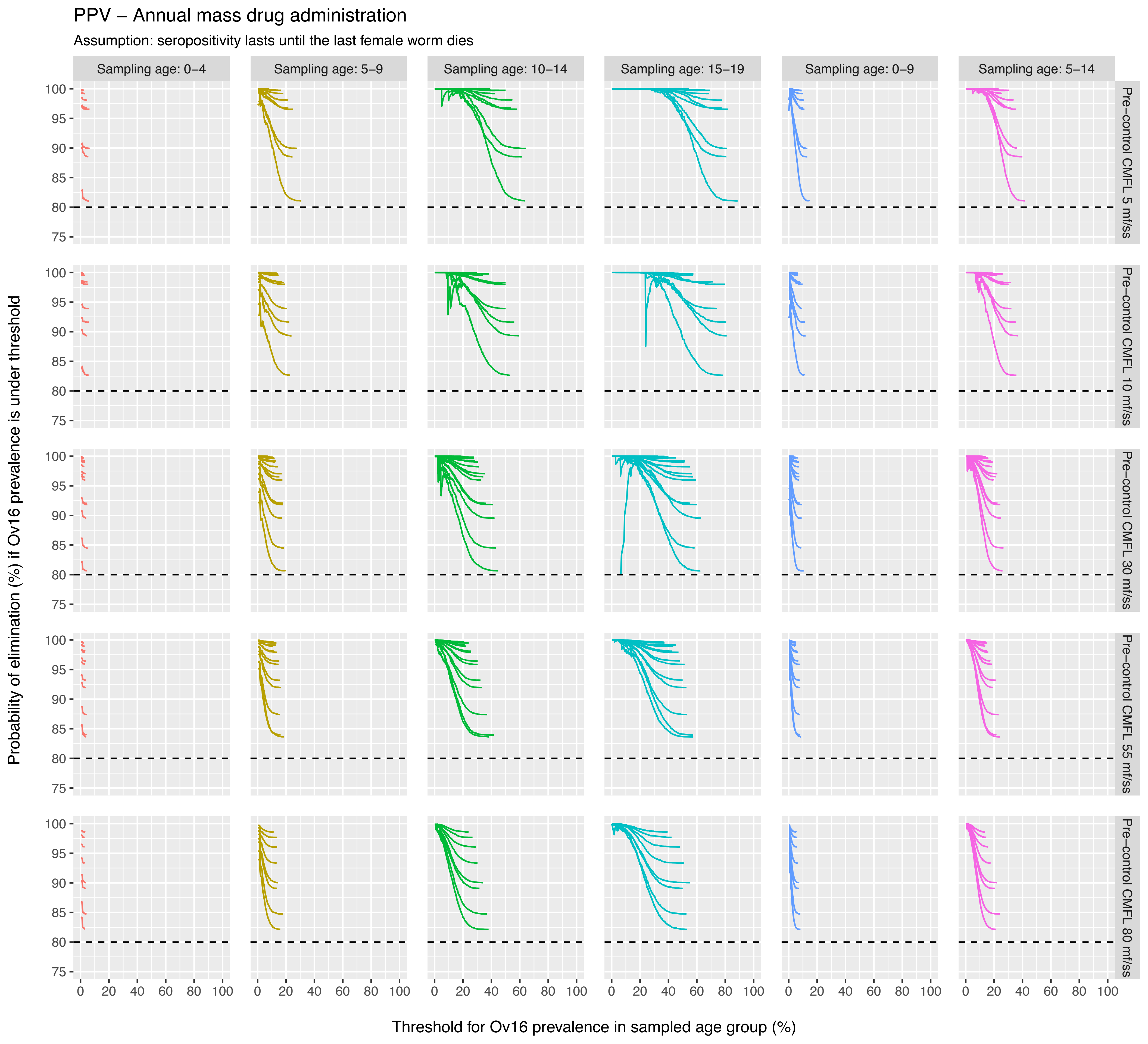

PPV – Annual mass drug administration
Assumption: seropositivity lasts until the last female worm dies
Sampling age: 0–4
Sampling age: 5–9
Sampling age: 10–14
Sampling age: 15–19
Sampling age: 0–9
Sampling age: 5–14
Pre–control CMFL 5 mf/ss
Pre–control CMFL 10 mf/ss
Pre–control CMFL 30 mf/ss
Pre–control CMFL 55 mf/ss
Pre–control CMFL 80 mf/ss
Probability of elimination (%) if Ov16 prevalence is under threshold
Threshold for Ov16 prevalence in sampled age group (%)

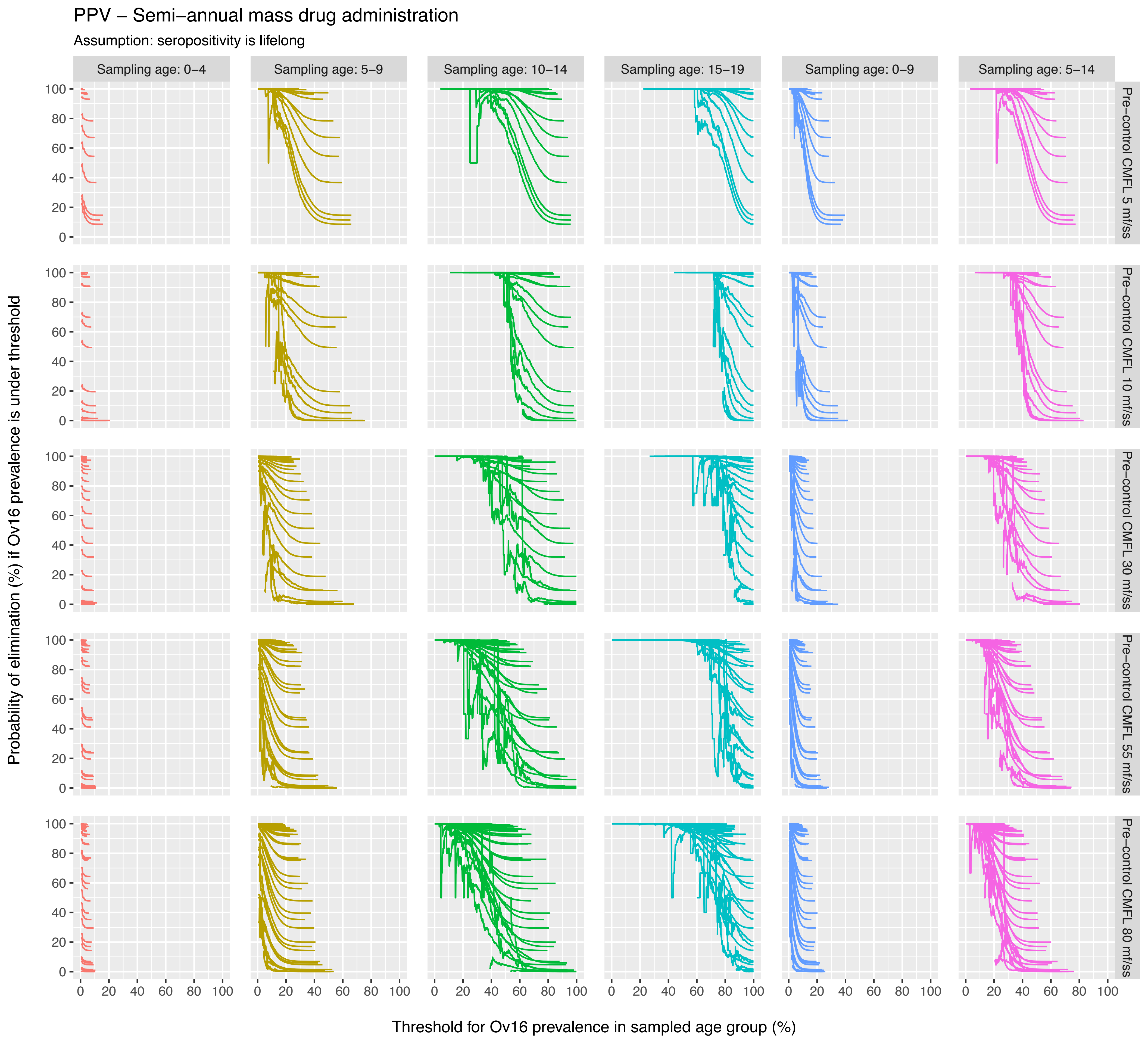
PPV – Semi–annual mass drug administration
Assumption: seropositivity is lifelong
Sampling age: 0–4
Sampling age: 5–9
Sampling age: 10–14
Sampling age: 15–19
Sampling age: 0–9
Sampling age: 5–14
Pre–control CMFL 5 mf/ss
Pre–control CMFL 10 mf/ss
Pre–control CMFL 30 mf/ss
Pre–control CMFL 55 mf/ss
Pre–control CMFL 80 mf/ss
Probability of elimination (%) if Ov16 prevalence is under threshold
Threshold for Ov16 prevalence in sampled age group (%)
0
20
40
60
80
100

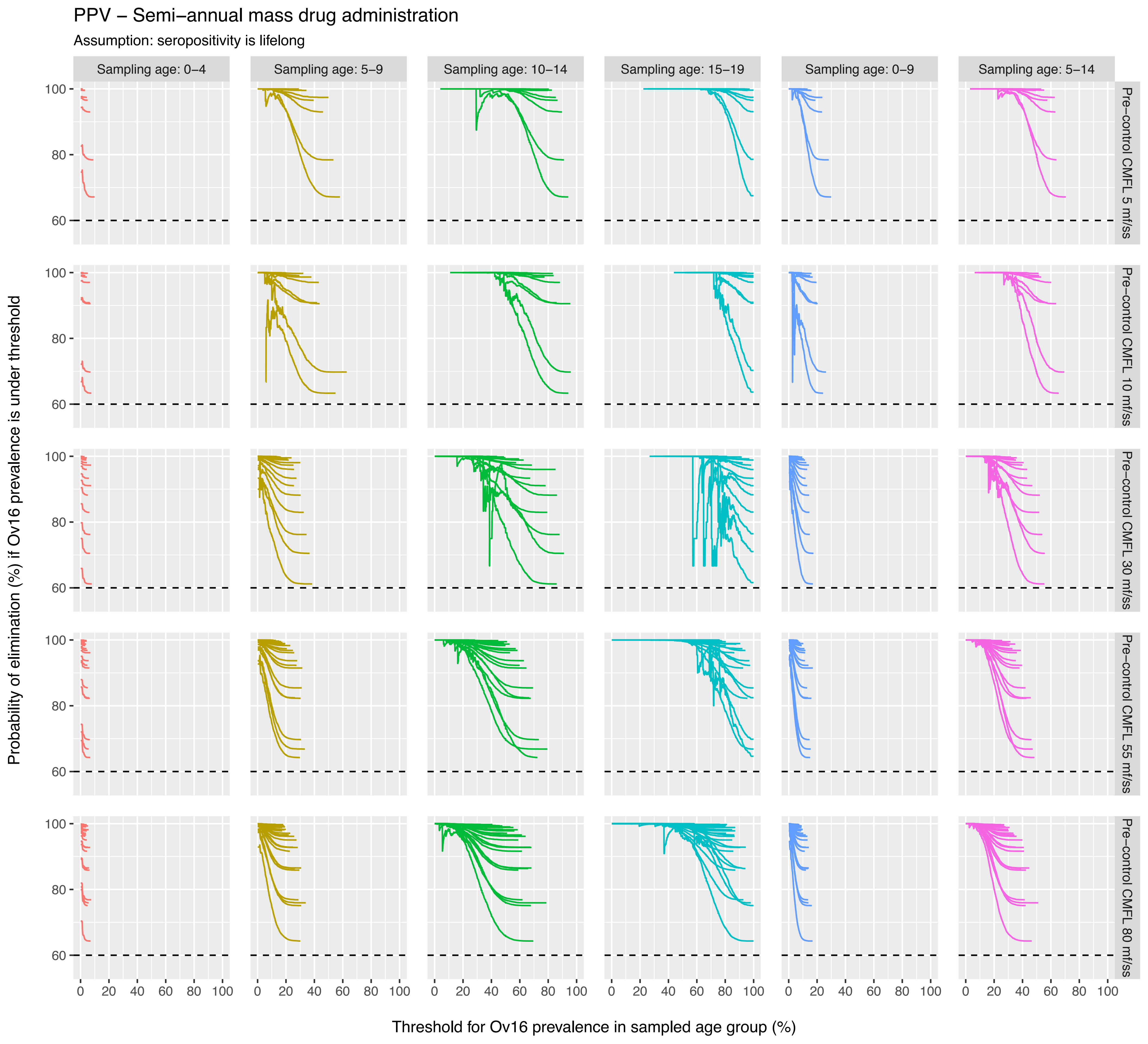

PPV – Semi–annual mass drug administration
Assumption: seropositivity is lifelong
Sampling age: 0–4
Sampling age: 5–9
Sampling age: 10–14
Sampling age: 15–19
Sampling age: 0–9
Sampling age: 5–14
Pre–control CMFL 5 mf/ss
Pre–control CMFL 10 mf/ss
Pre–control CMFL 30 mf/ss
Pre–control CMFL 55 mf/ss
Pre–control CMFL 80 mf/ss
Probability of elimination (%) if Ov16 prevalence is under threshold
Threshold for Ov16 prevalence in sampled age group (%)

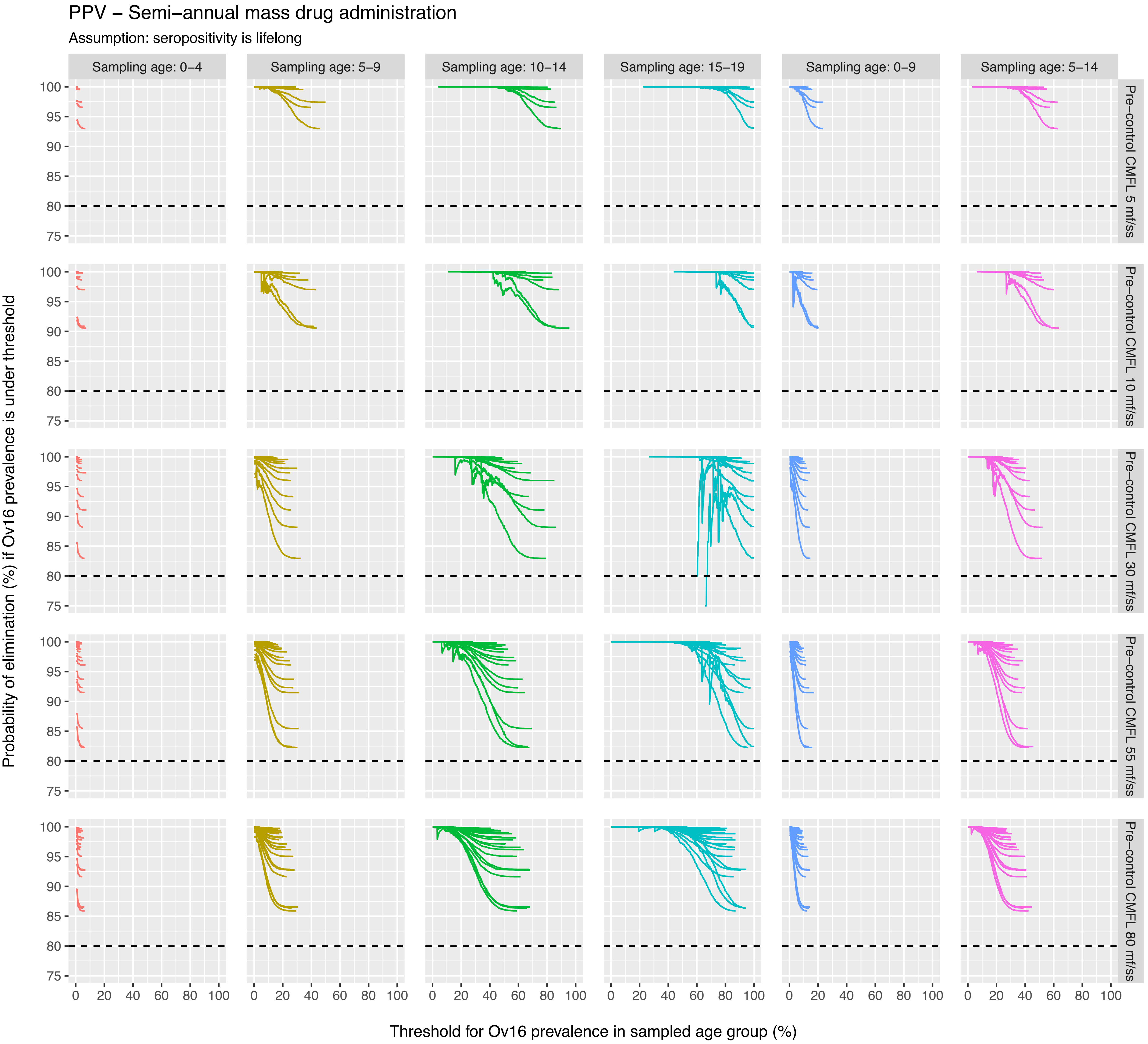
PPV – Semi–annual mass drug administration
Assumption: seropositivity is lifelong
Sampling age: 0–4
Sampling age: 5–9
Sampling age: 10–14
Sampling age: 15–19
Sampling age: 0–9
Sampling age: 5–14
Pre–control CMFL 5 mf/ss
Pre–control CMFL 10 mf/ss
Pre–control CMFL 30 mf/ss
Pre–control CMFL 55 mf/ss
Pre–control CMFL 80 mf/ss
Probability of elimination (%) if Ov16 prevalence is under threshold
Threshold for Ov16 prevalence in sampled age group (%)

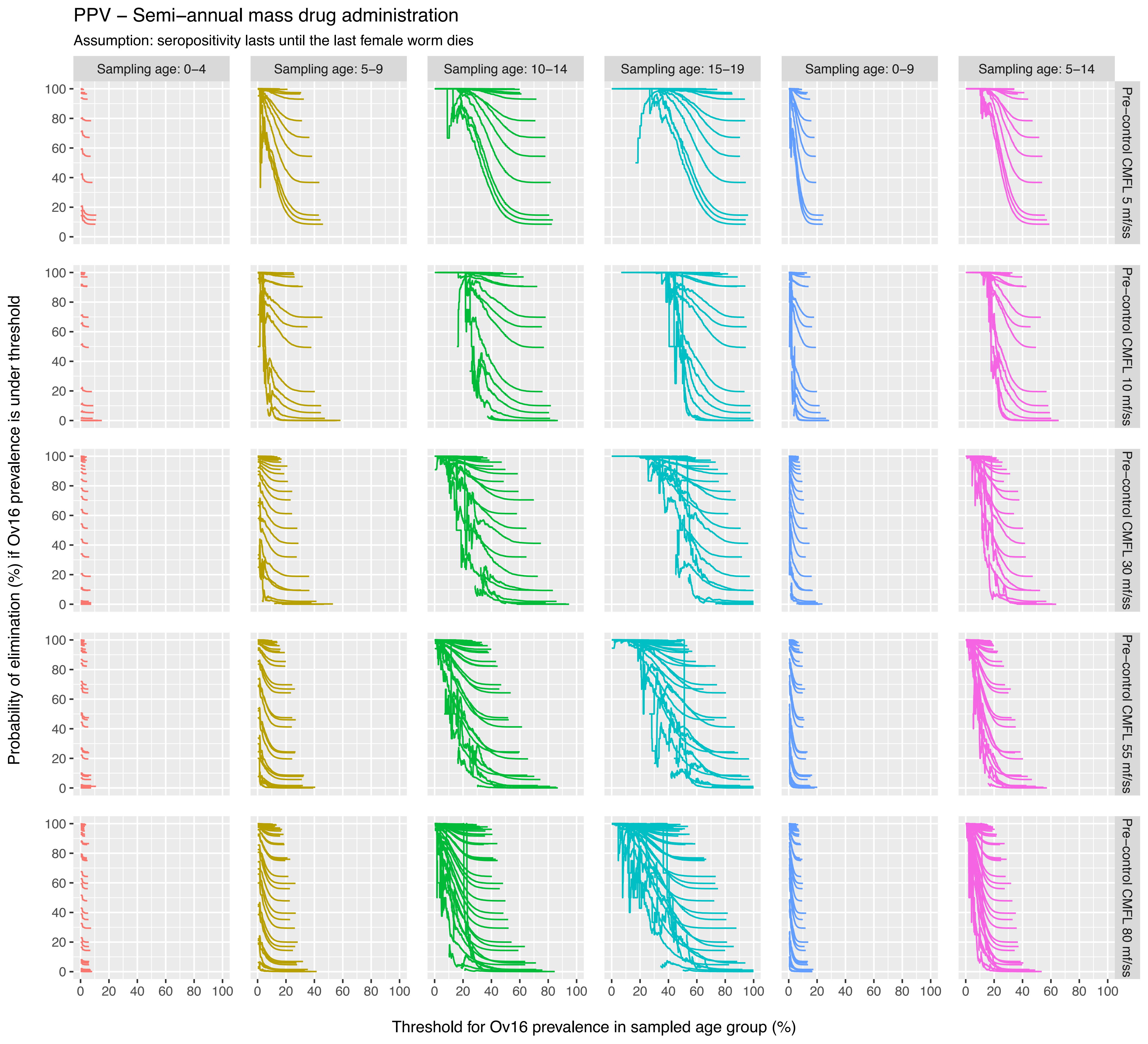
PPV – Semi−annual mass drug administration
Assumption: seropositivity lasts until the last female worm dies
Sampling age: 0−4
Sampling age: 5−9
Sampling age: 10−14
Sampling age: 15−19
Sampling age: 0−9
Sampling age: 5−14
Pre−control CMFL 5 mf/ss
Pre−control CMFL 10 mf/ss
Pre−control CMFL 30 mf/ss
Pre−control CMFL 55 mf/ss
Pre−control CMFL 80 mf/ss
Probability of elimination (%) if Ov16 prevalence is under threshold
Threshold for Ov16 prevalence in sampled age group (%)
0
20
40
60
80
100

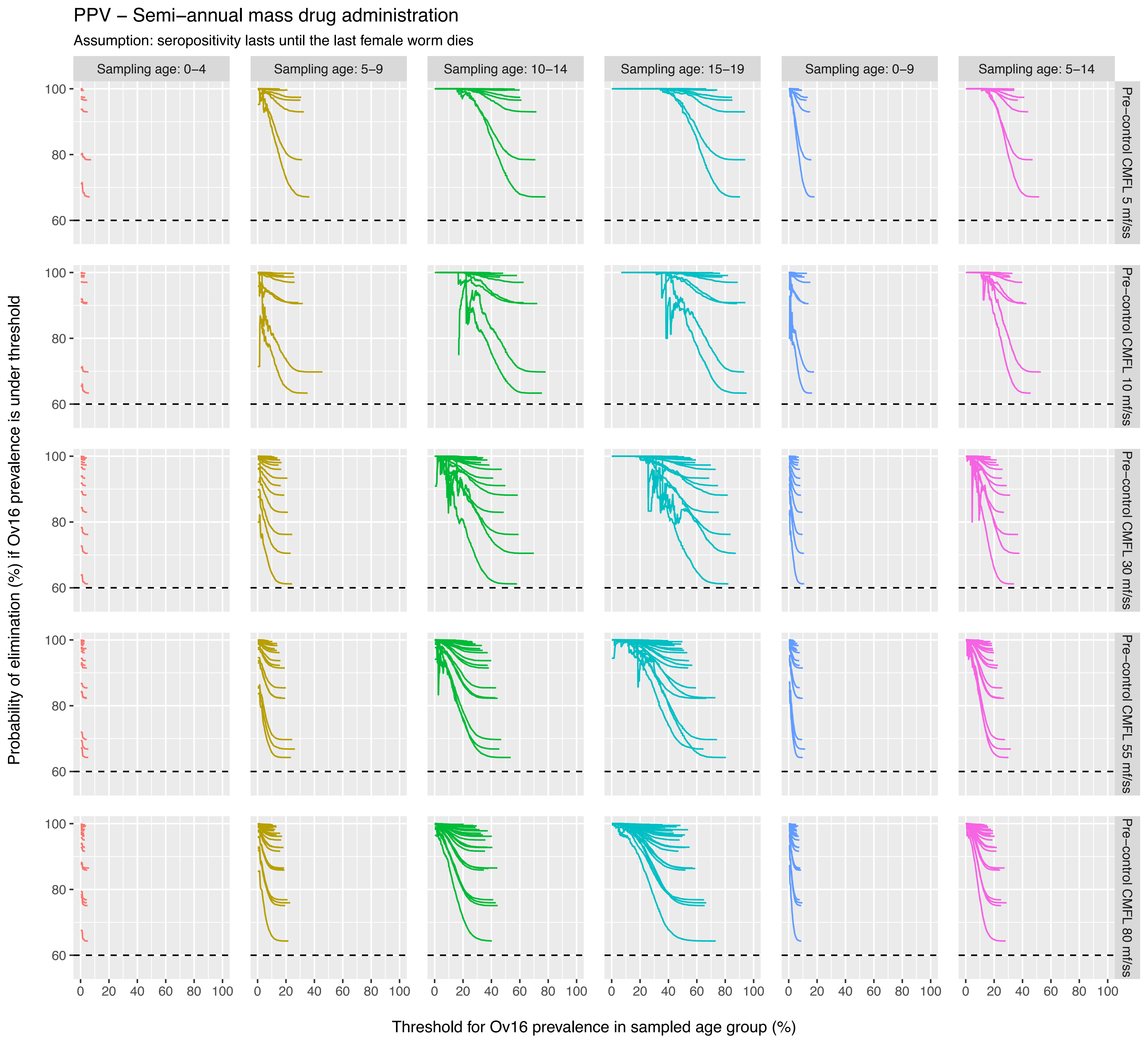

PPV – Semi–annual mass drug administration
Assumption: seropositivity lasts until the last female worm dies
Sampling age: 0–4
Sampling age: 5–9
Sampling age: 10–14
Sampling age: 15–19
Sampling age: 0–9
Sampling age: 5–14
Pre–control CMFL 5 mf/ss
Pre–control CMFL 10 mf/ss
Pre–control CMFL 30 mf/ss
Pre–control CMFL 55 mf/ss
Pre–control CMFL 80 mf/ss
Probability of elimination (%) if Ov16 prevalence is under threshold
Threshold for Ov16 prevalence in sampled age group (%)

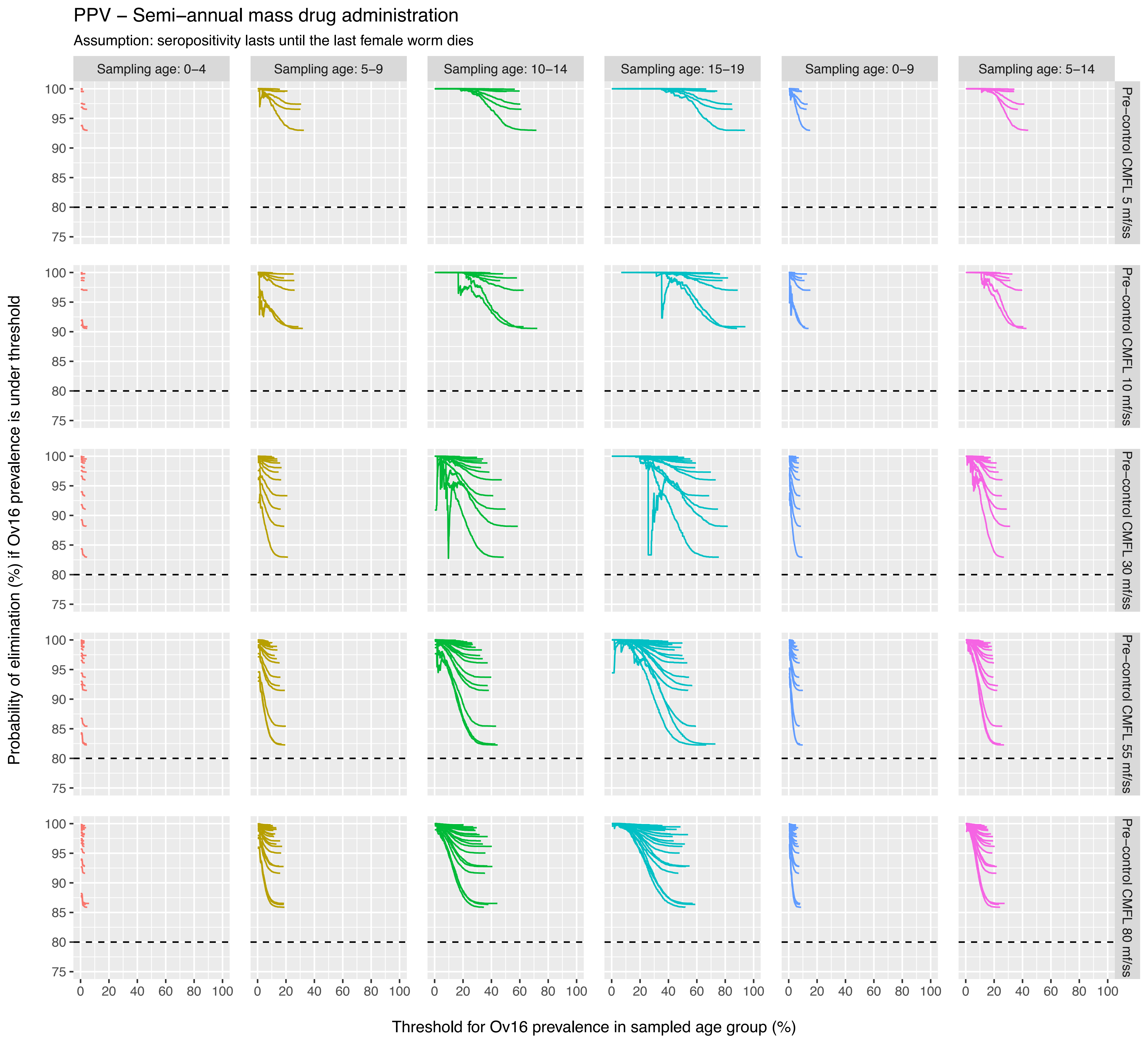
PPV – Semi–annual mass drug administration
Assumption: seropositivity lasts until the last female worm dies
Sampling age: 0–4
Sampling age: 5–9
Sampling age: 10–14
Sampling age: 15–19
Sampling age: 0–9
Sampling age: 5–14
Pre–control CMFL 5 mf/ss
Pre–control CMFL 10 mf/ss
Pre–control CMFL 30 mf/ss
Pre–control CMFL 55 mf/ss
Pre–control CMFL 80 mf/ss
Probability of elimination (%) if Ov16 prevalence is under threshold
Threshold for Ov16 prevalence in sampled age group (%)
